# Robustness of Topological Phases on Aperiodic Lattices

**DOI:** 10.1007/s11040-026-09547-1

**Published:** 2026-03-16

**Authors:** Yuezhao Li

**Affiliations:** Mathematical Institute, Universiteit Leiden, Einsteinweg 55, 2333 CC Leiden, The Netherlands

## Abstract

We study the robustness of topological phases on aperiodic lattices by constructing *-homomorphisms from the groupoid model to the coarse-geometric model of observable C*-algebras. These *-homomorphisms induce maps in K-theory and Kasparov theory. We show that the strong topological phases in the groupoid model are detected by position spectral triples. We show that topological phases coming from stacking along another Delone set are always weak in the coarse-geometric sense.

## Introduction

In this article, we aim at a new look at the following question:

How to understand the robustness of topological phases of a discrete physical system, described by a generic aperiodic point pattern?

We begin with some background around it. Topological insulators are materials that are insulating in the bulk but nevertheless possess metallic edges. The current flowing on the edge is usually quite robust under disorder coming from impurity of the crystal. Starting from Bellissard, van Elst and Schulz-Baldes [5, 11], the non-commutative and C*-algebraic tools have been intensively applied to the study of such materials and in particular, leading to an interpretation of the Kubo formula in the integer quantum Hall effect (IQHE) as a quantised index pairing.

In physics literature, the model Hamiltonian of a topological insulator is usually given by a tight-binding, short-range operator supported on a periodic, square lattice, invariant under the translation by its unit cell vector. Such a Hamiltonian $H$ (or its spectral projection) belong to a (noncommutative) torus $A_\vartheta =\mathbb {C}\rtimes _\vartheta \mathbb {Z}^d$, the noncommutativity coming from a 2-cocycle twist $\vartheta$ given by the external magnetic field. If the Hamiltonian is not translation invariant, then Bellissard suggested to replace $A_\vartheta$ by a twisted crossed product $\textrm{C}(\Omega )\rtimes _\vartheta \mathbb {Z}^d$, where $\Omega$ is a compact space called the hull of $H$. This description is applicable even if the underlying space is no longer a square lattice, but comes from a tiling subject to some properties, cf. [2, 27].

There are, however, amorphous materials, like liquid crystals and glass, that cannot be modelled using such methods. Even if for quasi-crystals such that the method of [2, 27] applies, then one has to essentially provide a (non-canonical) $\mathbb {Z}^d$-labelling of the underlying lattice, under which the Hamiltonian remains short-range (cf. [10]).

These suggest to use more general point sets in $\mathbb {R}^d$, modelling the atomic sites of this material, as their underlying geometric spaces. The dynamics thereon are studied by more general actions other than a $\mathbb {Z}^d$-translation. It was explained in [6] that such point sets should be *Delone sets* (Definition 3.1). Let $\Lambda$ be a Delone set, whose points are considered as the sites in a physical system. How should one model the observable C*-algebra $A$ from it?

The choice of an observable C*-algebra should be aligned with the following principle: it should be *large* enough to contain all possible Hamiltonians, and *small* enough to supply useful homotopy theory (K-theory). In recent years, there have been two main approaches to this modelling problem, which provide toolkits to compute invariants of topological phases:

- *“dynamical” approach* describes a crossed product C*-algebra, covariant for the *groupoid* actions on the aperiodic point pattern, then restricts to the dynamical hull of the point pattern. This gives a groupoid C*-algebra.
- *“coarse-geometric” approach* describes a C*-algebra which is stable under all *short-range*, *locally–finite-rank* perturbations that do not close the gap. This leads to a Roe C*-algebra.

The groupoid approach gives an étale groupoid $\mathcal {G}_\Lambda \rightrightarrows \Omega _0$ from a Delone set $\Lambda$, and yields a “tight-binding” groupoid C*-algebra $\textrm{C}^*(\mathcal {G}_\Lambda )$ , cf. [6, 9, 10, 23]. This is a generalisation of the well-studied periodic model: if $\Lambda =\mathbb {Z}^d$, then $\textrm{C}^*(\mathcal {G}_\Lambda )\simeq \textrm{C}^*(\mathbb {Z}^d)$ is the group C*-algebra of $\mathbb {Z}^d$. Here tight-binding means the following: the regular representation of $\textrm{C}^*(\mathcal {G}_\Lambda )$ is given by a Hilbert $\textrm{C}(\Omega _0)$-module, or equivalently, a continuous field of Hilbert spaces over $\Omega _0$, where $\Omega _0$ is unit space of the groupoid $\mathcal {G}_\Lambda$. The fibres of this continuous field are canonically identified with $\ell ^2(\omega )$, where $\omega \in \Omega _0$ is a Delone set, either as a translated copy of $\Lambda$, or as a weak*-limit of such sets. Thus $\textrm{C}^*(\mathcal {G}_\Lambda )$ consists of families of model Hamiltonians $(H_\omega )_{\omega \in \Omega _0}$, where $H_\omega$ acts on $\ell ^2(\omega )$, in a covariant way with respect to the groupoid action.

The coarse-geometric approach describes a C*-algebra which is stable under all possible short-range perturbations. This gives rise to a (uniform or non-uniform) Roe C*-algebra $\textrm{C}^*_\textrm{Roe}(\Lambda )$ from $\Lambda$, cf. [12, 19]. We choose to work with the non-uniform Roe C*-algebras, whose advantages over the uniform ones were explained in [12].

In this setup, a Delone set $\Lambda \subseteq \mathbb {R}^d$ is considered as a discrete metric space. Then $L^2(\mathbb {R}^d)$ carries an ample representation of $\textrm{C}_0(\Lambda )$, which generates a Roe C*-algebra $\textrm{C}^*_\textrm{Roe}(\Lambda )$. Physically, a short-range Hamiltonian $H$ has matrix coeffcients  of fast enough decay, thus can be approximated by controlled operators. Every unit cell should have finite degrees of freedom, coming from the number of electron orbits and their spins. Thus the restriction of $H$ to any finite region should have finite-rank. Therefore, the Roe C*-algebra $\textrm{C}^*_\textrm{Roe}(\Lambda )$ can be viewed as the universal C*-algebra that contains all such Hamiltonians.

If we model a topological insulator on a Delone set $\Lambda$ by the Roe C*-algebra $\textrm{C}^*_\textrm{Roe}(\Lambda )$, then its topological phase is robust in a very strong sense: such topological phases (and their associated topological invariants) are robust under any short-range, locally–finite-rank perturbation that preserves the spectral gap and the symmetry of the system. It follows from the K-theory of (real) Roe C*-algebra (cf. (3.24)) that such topological phase are completely classified by their topological invariants, which are $\mathbb {Z}$- or $\mathbb {Z}/2$-valued indiced that belong to the real K-theory groups of $\mathbb {R}$. The groupoid model $\textrm{C}^*(\mathcal {G}_\Lambda )$, on the other hand, has more involved K-theory and numerical invariants. One natural question is in which sense those topological phases described by the groupoid model are still robust. Moreover, do they lead to interesting invariants that do not occur in the coarse-geometric model?

Questions related to this type of robustness are usually answered by showing that the numerical index is still continuous, even for some more general perturbations of the Hamiltonian (which does not have to belong to the observable C*-algebra), together with a “quantisation” statement that the range of the numerical index is $\mathbb {Z}$. This implies that such perturbations leave the topological invariants unchanged.

We describe a different approach to this robustness question by comparing the groupoid model with the coarse-geometric model on $\Lambda$ using the regular representation. The topological invariants defined in [9] factor through this representation. Therefore, since $\textrm{C}^*_\textrm{Roe}(\Lambda )$ is “robust”, one only needs to understand which topological phases in $\textrm{C}^*(\mathcal {G}_\Lambda )$ may still survive there. To this end, we introduce a handy definition of position spectral triples (Definition 2.11). Both the groupoid model and the coarse-geometric model possess such type of spectral triples. In particular, the position spectral triple $\xi ^\textrm{Roe}_\omega$ over the Roe C*-algebra $\textrm{C}^*_\textrm{Roe}(\omega )$ induces an isomorphism $\operatorname {KK}(\textrm{C}\ell _{d,0},\textrm{C}^*_\textrm{Roe}(\omega ))\rightarrow \mathbb {Z}$ (Theorem 3.33). Then we show that those topological invariants of the groupoid model, given by localising the “bulk cycle” $_{d}{\lambda }_{\Omega _0}$ at $\omega \in \Omega _0$, are pullbacks of the KK-class of $\xi ^\textrm{Roe}_\omega$ under the comparison *-homomorphism $\mathbb {M}_N(\textrm{C}^*(\mathcal {G}_\Lambda ))\rightarrow \textrm{C}^*_\textrm{Roe}(\omega )$ (Theorem 4.1). Such invariants are therefore strong in the sense of [12].

As another application, we show in Theorem 4.10 that certain numerical invariants on Delone sets, coming from “stacking” lower-dimensional topological phases, must be weak, i.e. unstable under perturbation. This generalises a result in [12], which explains why certain topological phases of the periodic model ought to be weak. More precisely, the authors of [12] compare the periodic model and the coarse-geometric model using an injective *-homomorphism $\textrm{C}^*(\mathbb {Z}^d)\hookrightarrow \textrm{C}^*_\textrm{Roe}(\mathbb {Z}^d)$. It vanishes on all but one $\mathbb {Z}$-component of K-theory. In particular, topological phases coming from “stacking” lower-dimensional topological phases will all vanish in $\operatorname {K}_*(\textrm{C}^*_\textrm{Roe}(\mathbb {Z}^d))$. This allows us to conclude that the induced maps $\operatorname {K}_*(\textrm{C}^*(\mathbb {Z}^{d}))\rightarrow \operatorname {K}_*(\textrm{C}^*(\mathbb {Z}^{d+1}))$ also vanishes there. We show that one can replace $\mathbb {Z}^{d+1}$ by a product Delone set of the form $\Lambda \times L$, where $\Lambda \subseteq \mathbb {R}^p$ and $L\subseteq \mathbb {R}^q$ are both Delone sets. Using the same strategy in [12], we show that stacked topological phases factor through the Roe C*-algebra of a flasque space, which has vanishing K-theory. This allows us to conclude that such topological phases and their numerical invariants have to vanish in the target Roe C*-algebra.

### Notation and Conventions

We fix the following symbols and conventions.

*Dirac notation* Let $\Lambda$ be a discrete set. We write $|x\rangle$ for the function in $\ell ^2(\Lambda )$ which takes $1$ on $x$ and $0$ elsewhere; and $\langle x|$ for the rank-one operator $\ell ^2(\Lambda )\rightarrow \mathbb {C}$ defined by $|y\rangle \mapsto \left\langle x,y\right\rangle$. Let $T\in \mathbb {B}(\ell ^2(\Lambda ))$, then by  we shall mean the inner product of $\left|x\right\rangle$ and $T\left|y\right\rangle$. The collection $(T_{x,y})_{x,y\in \Lambda }$ of  are called the *matrix elements* of $T$.

*Tensor products* Let $A$ and $B$ be $\mathbb {Z}/2$-graded “real”C*-algebras. We will write $A \otimes B$ for their *graded*, minimal tensor product. The grading will cause little difference in this article: we will mostly consider $\mathbb {Z}/2$-graded C*-algebras of the form $A\otimes \mathbb {C}\ell _{p,q}$ or $A\otimes \textrm{C}\ell _{p,q}$ for a trivial graded (real or complex) C*-algebra $A$. In such cases, the graded tensor product agrees with the ungraded version.

*Group*(*oid*)* and Roe *C*-algebras We write $\textrm{C}^*(\mathcal {G})$ for the reduced C*-algebra of a groupoid or a group, and $\textrm{C}^*_\textrm{Roe}(X,\mathcal {H}_X)$ for the Roe C*-algebra defined by an ample module $(X,\mathcal {H}_X)$. This is aligned with the convention in [12]. As a potentially confusing point, we note that $\textrm{C}^*(\mathbb {Z}^d)$ is the *group* C*-algebra of the discrete group $\mathbb {Z}^d$, and $\textrm{C}^*_\textrm{Roe}(\mathbb {Z}^d)$ is the *Roe* C*-algebra of the discrete metric space $\mathbb {Z}^d$.

## Topological Phases and K-Theory

In the simplest form, a (non-interacting) quantum system is described by an unbounded, self-adjoint operator $H$ (the Hamiltonian) acting on a complex, separable Hilbert space $\mathcal {H}$. A *topological insulator* is a quantum system which carries certain symmetries, giving rise to *topological invariants* that distinguish different *topological phases* of this system.

The links between topological insulators and (real, bivariant) K-theory has been investigated throughly in the recent decade, cf. [4, 17, 28]. In our case of interest, the links are made as follows:

1. For aperiodic materials, the physically relevant symmetries are time-reversal symmetry, particle-hole symmetry and chiral symmetry. These symmetries may be combined and give rise to several symmetry types of the system. The topological phase of such a material is represented by the class $[H]\in \operatorname {KK}(\textrm{C}\ell _{n,0},A)$ of the Hamiltonian in a certain bivariant K-theory (in fact, $\operatorname {KO}$-theory) group of a real C*-algebra $A$ (the observable real C*-algebra), cf. [ [28], Table 1; [4], Table 1; [17], Section 6].
2. The topological invariant of a topological insulator can be computed as an *index pairing*. That is, a *Kasparov product* of the form
  $$\begin{aligned} \operatorname {KK}(\textrm{C}\ell _{n,0},A)\times \operatorname {KK}(A\otimes \textrm{C}\ell _{0,d},\mathbb {R})\rightarrow \operatorname {KK}(\textrm{C}\ell _{n,d},\mathbb {R})\simeq \operatorname {KO}_{n-d}(\mathbb {R}), \end{aligned}$$
  where
  $$\begin{aligned} \operatorname {KO}_{n-d}(\mathbb {R})\simeq {\left\{ \begin{array}{ll} \mathbb {Z}& \text {if }n-d\equiv 0,4\,\,\,\!\mod 8; \\ \mathbb {Z}/2 & \text {if }n-d\equiv 1, 2\,\,\,\!\mod 8; \\ 0 & \text {if }n-d\equiv 3, 5, 6, 7\!\mod 8 \end{array}\right. } \end{aligned}$$
  is the receptacle of $\mathbb {Z}$- or $\mathbb {Z}/2$-valued indices that can be measured in physical experiments.

The section is organised as follows. We shall first recall in Section 2.1 the definitions of “real” and real C*-algebras and their representations. Then we study two special cases: the Clifford algebras in Section 2.1.1 and the graded “real” C*-algebra $\textrm{C}^*(\mathbb {Z}^d)\otimes \mathbb {C}\ell _{0,d}$ in Section 2.1.2. We recall a few properties of Kasparov theory in Section 2.2 that will be used later.

We introduce a class of spectral triples, called position spectral triples, in Section 2.3. We recall in Section 2.3.1 that the position spectral triple $\xi _{\mathbb {Z}^d}$ over $\textrm{C}^*(\mathbb {Z}^d)_\mathbb {R}\otimes \textrm{C}\ell _{0,d}$ represents the fundamental class. Via the Kasparov product, it generates a surjective group homomorphism

$$\begin{aligned} [\xi ]:\operatorname {KK}(\textrm{C}^*(\mathbb {Z}^d)_\mathbb {R}\otimes \textrm{C}\ell _{0,d})\rightarrow \operatorname {KK}(\textrm{C}\ell _{d,d},\mathbb {R})\simeq \mathbb {Z}. \end{aligned}$$

### “Real” and Real C*-Algebras

When we consider physical systems with anti-unitary symmetries, like time-reversal or particle-hole symmetries, then we must represent them as anti-unitary operators on $\mathcal {H}$ which commute or anti-commute with the Hamiltonian $H$. This turns the Hilbert space $\mathcal {H}$ into a “real” Hilbert space $(\mathcal {H},\Theta )$, and the observable C*-algebra into a “real” C*-algebra $(A,\mathfrak {r})$. The latter was introduced by Kasparov ( [14], Section 1, Definition 3).

We recall the definition of “real” and real C*-algebras and Hilbert spaces. A “real” C*-algebra is sometimes referred to as a Real C*-algebra (cf. [7], note the upper case R) or a $\textrm{C}^{*,\textrm{r}}$-algebra (cf. [17]) in the literature.

#### Definition 2.1

([17], Definition 3.7) A “real” structure on a $\mathbb {Z}/2$-graded C*-algebra $A$ is a conjugate-linear, grading-preserving *-automorphism $\mathfrak {r}:A\rightarrow A$ of order 2. A “real” C*-algebra is a C*-algebra together with a “real” structure on it.

A “real” involution on a $\mathbb {Z}/2$-graded Hilbert space $\mathcal {H}$ is a conjugate-linear, grading-preserving automorphism $\Theta :\mathcal {H}\rightarrow \mathcal {H}$ of order two. A “real” Hilbert space is a Hilbert space $\mathcal {H}$ together with a “real” involution $\Theta$ on it.

A representation of a $\mathbb {Z}/2$-graded “real” C*-algebra $A$ is a *-homomorphism $\pi :A\rightarrow \mathbb {B}(\mathcal {H})$ for a $\mathbb {Z}/2$-graded “real” Hilbert space $\mathcal {H}$, which intertwines both the $\mathbb {Z}/2$-gradings and the “real” structures.

Let $\Theta$ be a “real” involution on a $\mathbb {Z}/2$-graded Hilbert space. Then $\mathbb {B}(\mathcal {H})$ becomes a $\mathbb {Z}/2$-graded “real” C*-algebra with “real” structure $T\mapsto \Theta T\Theta ^*$. A (possibly unbounded) operator on $\mathcal {H}$ is said to be “real” if it commutes with $\Theta$.

Now we recall real C*-algebras and real Hilbert spaces.

#### Definition 2.2

A real Hilbert space is a Hilbert space over $\mathbb {R}$. A real C*-algebra is a norm-closed subalgebra of $\mathbb {B}(\mathcal {H_\mathbb {R}})$, where $\mathcal {H_\mathbb {R}}$ is a Hilbert space over $\mathbb {R}$.

If $(A,\mathfrak {r})$ is a “real” C*-algebra, then the fixed points of its real structure

$$\begin{aligned}A^{\mathfrak {r}}\mathrel {:=}\left\rbrace a\in A\;\big |\;\mathfrak {r}(a)=a\right\lbrace \end{aligned}$$

is a real C*-algebra. Conversely, if $A_\mathbb {R}$ is a real C*-algebra, then $A_\mathbb {R}\otimes _\mathbb {R}\mathbb {C}$ is a “real” C*-algebra with “real” structure

$$\begin{aligned}\mathfrak {r}(a\otimes _\mathbb {R}z)\mathrel {:=}a\otimes _\mathbb {R}\overline{z}. \end{aligned}$$

Let $(X,\tau )$ be an locally compact, Hausdorff involutive space, that is, a locally compact Hausdorff space $X$ together with a homeomorphism $\tau :X\rightarrow X$ satisfying $\tau ^2=\operatorname {id}_X$. If $X$ is a manifold, then we also say $(X,\tau )$ is a “real” manifold. The C*-algebra $\textrm{C}_0(X)$ carries a “real” structure

$$\begin{aligned}\mathfrak {r}(f)(x)\mathrel {:=}\overline{f(\tau (x))}, \end{aligned}$$

yielding a “real” C*-algebra $(\textrm{C}_0(X),\mathfrak {r})$ as well as its corresponding real C*-algebra $\textrm{C}_0(X)^\mathfrak {r}$. The “real” Gelfand–Naimark theorem due to Arens and Kaplansky ( [1], Theorem 9.1) asserts that every commutative “real” C*-algebra is isomorphic to $(\textrm{C}_0(X),\mathfrak {r})$ for some involutive space $(X,\tau )$; and every commutative real C*-algebra is isomorphic to $\textrm{C}_0(X)^\mathfrak {r}$.

#### Clifford Algebras

Let $\mathbb {C}\ell _{p,q}$ be the finite-dimensional $\mathbb {Z}/2$-graded “real” C*-algebra generated by $\gamma ^1,\dots ,\gamma ^p,\rho ^1,\dots ,\rho ^q$, satisfying:

- $\gamma ^1,\dots ,\gamma ^p$ are odd, self-adjoint, involutive and real;
- $\rho ^1,\dots ,\rho ^q$ are odd, anti-self-adjoint, anti-involutive and real;
- $\gamma ^1,\dots ,\gamma ^p,\dots ,\rho ^1,\dots ,\rho ^q$ mutually anti-commute.

That is, we require that for all $i,j$:

$$\begin{aligned}&(\gamma ^j)^2=1,\quad  &   (\gamma ^j)^*=\gamma ^j,\quad  &   \mathfrak {r}(\gamma ^j)=\gamma ^j; \\&(\rho ^j)^2=-1,\quad  &   (\rho ^j)^*=-\rho ^j,\quad  &   \mathfrak {r}(\rho ^j)=\rho ^j. \end{aligned}$$

The real subalgebra of $\mathbb {C}\ell _{p,q}$ is the $\mathbb {R}$-algebra generated by the same generators and relations. We write $\textrm{C}\ell _{p,q}\mathrel {:=}(\mathbb {C}\ell _{p,q})^\mathfrak {r}$ for this $\mathbb {Z}/2$-graded, real C*-algebra. Up to Morita equivalence of graded real C*-algebras, there are only eight possible $\textrm{C}\ell _{p,q}$ satisfying

$$\begin{aligned} \textrm{C}\ell _{p,q}\otimes \textrm{C}\ell _{p',q'}\simeq \textrm{C}\ell _{p+p',q+q'}. \end{aligned}$$

Moreover, $\textrm{C}\ell _{1,1}$ is isomorphic to the $\mathbb {Z}/2$-graded real C*-algebra $\mathbb {M}_2(\mathbb {R})$, whose grading is given by diagonal–off-diagonal elements. Thus

$$\begin{aligned} \textrm{C}\ell _{d,d}\simeq \mathbb {M}_2(\mathbb {R})\otimes \mathbb {M}_2(\mathbb {R})\otimes \dots \otimes \mathbb {M}_2(\mathbb {R}) \simeq \mathbb {M}_{2^d}(\mathbb {R}). \end{aligned}$$

Kasparov has constructed a canonical representation of $\mathbb {C}\ell _{p,q}$ in [15]. Let $\mathbb {C}^d$ be the “real” Hilbert space with basis $e_1,\dots ,e_d$ and “real” involution

$$\begin{aligned} \sum _{i=1}^dc_ie_i\;\longmapsto \;\sum _{i=1}^d\overline{c_i}e_i. \end{aligned}$$

Let $\bigwedge \nolimits ^*\mathbb {C}^{d}$ be the exterior algebra of $\mathbb {C}^d$. It is graded by the subspace of odd or even differential forms $\bigwedge \nolimits ^*\mathbb {C}^{d}=\bigwedge \nolimits ^{\text {odd}}\mathbb {C}^d\oplus \bigwedge \nolimits ^{\text {even}}\mathbb {C}^d$. The “real” structure on $\mathbb {C}^d$ extends to $\bigwedge \nolimits ^*\mathbb {C}^{d}$, that is,

$$\begin{aligned} \sum _{i_ii_2\dots i_k}a_{i_1i_2\dots i_k}e_{i_1}\wedge e_{i_2}\wedge \dots \wedge e_{i_k}\longmapsto \sum _{i_ii_2\dots i_k}\overline{a_{i_1i_2\dots i_k}}e_{i_1}\wedge e_{i_2}\wedge \dots \wedge e_{i_k}, \end{aligned}$$

turning $\bigwedge \nolimits ^*\mathbb {C}^{d}$ into a $\mathbb {Z}/2$-graded “real” Hilbert space.

##### Definition and Lemma 2.3

Let $j\in \{1,\dots ,d\}$ and $\lambda _j:\bigwedge \nolimits ^*\mathbb {C}^{d}\rightarrow \bigwedge \nolimits ^*\mathbb {C}^{d}$ be the exterior product with $e_j$, that is, $\lambda _j(\omega )=e_j\wedge \omega$. Then its adjoint $\lambda _j^*:\bigwedge \nolimits ^*\mathbb {C}^{d}\rightarrow \bigwedge \nolimits ^*\mathbb {C}^{d}$ is contraction with $e_j$, that is, $\lambda _j^*(\omega )=e_j\mathbin {\lrcorner }\omega$.

As a consequence, there is a representation of the $\mathbb {Z}/2$-graded “real” C*-algebra $\mathbb {C}\ell _{p,q}$ on $\bigwedge \nolimits ^*\mathbb {C}^{p+q}$, sending $\gamma ^j$ to $\lambda _j+\lambda _j^*$ and $\rho ^j$ to $\lambda _{p+j}-\lambda _{p+j}^*$. We call it the standard representation of $\mathbb {C}\ell _{p,q}$.

##### Proof

The operators $\lambda _j$ and their adjoints satisfy

$$\begin{aligned} \lambda _i\lambda _j+\lambda _j\lambda _i=0,\quad \lambda _i^*\lambda _j+\lambda _j\lambda _i^*=\left\langle e_i,e_j\right\rangle . \end{aligned}$$

Moreover, $$ \lambda _j $$ and $$ \lambda _j^* $$ are odd and real for all $$ j$$. So we have

$$\begin{aligned} (\lambda _j+\lambda _j^*)^2=1,\quad (\lambda _j-\lambda _j^*)^2=-1,\quad (\lambda _i+\lambda _i^*)(\lambda _{p+j}-\lambda _{p+j}^*)=0. \end{aligned}$$

Thus the operators $\lambda _1+\lambda _1^*,\dots \lambda _r+\lambda _r^*,\dots ,\lambda _{p+1}-\lambda _{p+1}^*,\dots \lambda _{p+q}-\lambda _{p+q}^*$ generate a copy of $\mathbb {C}\ell _{p+q}$ in $\mathbb {B}(\bigwedge \nolimits ^*\mathbb {C}^{p+q})$. $\square$

#### The $\mathbb {Z}/2$-graded “real” C*-algebra $\textrm{C}^*(\mathbb {Z}^d)\otimes \mathbb {C}\ell _{0,d}$

Next, we describe a representation of the graded “real” C*-algebra $\textrm{C}^*(\mathbb {Z}^d)\otimes \mathbb {C}\ell _{0,d}$ following ( [12], Section 4). The grading on $\textrm{C}^*(\mathbb {Z}^d)\otimes \mathbb {C}\ell _{0,d}$ is the tensor product of the trivial grading on $\textrm{C}^*(\mathbb {Z}^d)$ with the standard grading on $\mathbb {C}\ell _{0,d}$. The “real” structure on $\textrm{C}^*(\mathbb {Z}^d)$ is given by the pointwise complex conjugation, that is,

$$\begin{aligned} \mathfrak {r}(f)(n)\mathrel {:=}\overline{f(n)},\quad f\in \textrm{C}_\textrm{c}(\mathbb {Z}^d),\;n\in \mathbb {Z}^d. \end{aligned}$$

Then the regular representation of the complex C*-algebra $\textrm{C}^*(\mathbb {Z}^d)$ extends to a *-representation on the “real” Hilbert space $\ell ^2(\mathbb {Z}^d)$, whose “real” structure is given by pointwise conjugation. The “real” structure on $\textrm{C}^*(\mathbb {Z}^d)\otimes \mathbb {C}\ell _{0,d}$ is the tensor product of this “real” structure with the standard one on $\mathbb {C}\ell _{0,d}$.

The Fourier transform maps the “real” C*-algebra $\textrm{C}^*(\mathbb {Z}^d)$ to the “real” C*-algebra $\textrm{C}(\mathbb {T}^d)$. Here $\mathbb {T}^d$ is a “real” $d$-torus, with involution $z\mapsto \overline{z}$.

##### Lemma 2.4

(cf. ( [12], Section 4))

There is an isomorphism of $\mathbb {Z}/2$-graded, “real” Hilbert spaces

2.5 $$\begin{aligned} U:\ell ^2(\mathbb {Z}^d)\otimes \bigwedge \nolimits ^*\mathbb {C}^{d}\xrightarrow {\sim }L^2\left( \bigwedge \nolimits ^*\textrm{T}^*\mathbb {T}^d\right) \end{aligned}$$

given by

$$\begin{aligned} \left|k\right\rangle \otimes e_{i_1}\wedge \dots \wedge e_{i_l}\mapsto \frac{z^k}{z_{i_1}\dots z_{i_l}}\textrm{d}z_{i_1}\wedge \dots \wedge \textrm{d}z_{i_l}, \end{aligned}$$

where

$$\begin{aligned} z^k\mathrel {:=}z_1^{k_1}z_2^{k_2}\dots z_d^{k_d},\quad k=(k_1,\dots ,k_d)\in \mathbb {Z}^d. \end{aligned}$$

This induces an isomorphism of $\mathbb {Z}/2$-graded “real” C*-algebras

$$\begin{aligned} \textrm{C}^*(\mathbb {Z}^d)\otimes \mathbb {C}\ell _{0,d}\simeq \textrm{C}(\mathbb {T}^d)\otimes \mathbb {C}\ell _{0,d}\simeq \textrm{C}(\mathbb {T}^d,\mathbb {C}\ell (\mathbb {T}^d)) \end{aligned}$$

where $\mathbb {C}\ell (\mathbb {T}^d)$ is the “real” Clifford bundle of the “real” manifold $\mathbb {T}^d$. In particular, under this isomorphism, the canonical representation of $\mathbb {C}\ell _{0,d}$ as in Definition and Lemma 2.3 is mapped to the Clifford multiplication of $\mathbb {C}\ell (\mathbb {T}^d)$ on $L^2\left( \bigwedge \nolimits ^*\textrm{T}^*\mathbb {T}^d\right)$.

We note that the “real” structure $\mathfrak {r}$ on the “real” C*-algebra $\textrm{C}(\mathbb {T}^d)$ induced by the involution $z\mapsto \overline{z}$ is given by

$$\begin{aligned} (\mathfrak {r}f)(z)\mathrel {:=}\overline{f(\overline{z})}, \end{aligned}$$

thus the fixed point algebra is

$$\begin{aligned} \text {C}(\mathbb {T}^d)^\mathfrak {r}\mathrel {:=}\left\{ f\in \text {C}(\mathbb {T}^d)\;|\;\ \overline{f(z)}= f{(\overline{z})}\right\} . \end{aligned}$$

Under the Fourier transform, $\textrm{C}(\mathbb {T}^d)^\mathfrak {r}$ is identified with the real group C*-algebra $\textrm{C}^*(\mathbb {Z}^d)_\mathbb {R}$, cf. Section 2.3.1.

Let  be the $j$-th position operator on $\ell ^2(\mathbb {Z}^d)$ defined byThus the operator  is an odd, essentially self-adjoint operator on the $\mathbb {Z}/2$-graded “real” Hilbert space $\ell ^2(\mathbb {Z}^d)\otimes \bigwedge \nolimits ^*\mathbb {C}^{d}$.

##### Lemma 2.6

The Fourier transform (2.5) induces a unitary equivalence between the unbounded operator  on $\ell ^2(\mathbb {Z}^d)\otimes \bigwedge ^*\mathbb {C}^d$ and the Hodge–de Rham operator $\textrm{d}+\textrm{d}^*$ on $L^2(\bigwedge ^*\textrm{T}^*\mathbb {T}^d)$. Both operators preserve the real subspace $\ell ^2(\mathbb {Z}^d)_\mathbb {R}\otimes \bigwedge \nolimits ^*\mathbb {R}^{d}\simeq L^2(\bigwedge ^*\textrm{T}^*\mathbb {T}^d)_\mathbb {R}$, and hence are “real”.

##### Proof

By definition, we haveThe de Rham operator $\textrm{d}$ satisfies

$$\begin{aligned} \textrm{d}\left( \frac{z^k}{z_{i_1}\dots z_{i_l}}\textrm{d}z_{i_1}\wedge \dots \wedge \textrm{d}z_{i_l}\right)&=\sum _{j=1}^d z_1^{k_1}\cdots (k_jz_j^{k_j-1}\textrm{d}z_j)\cdots z_d^{k_d}\wedge \frac{\textrm{d}z_{i_1}}{z_{i_1}}\wedge \dots \wedge \frac{\textrm{d}z_{i_l}}{z_{i_l}}. \\&=\sum _{j=1}^dk_jz^k\cdot \frac{\textrm{d}z_j}{z_j}\wedge \frac{\textrm{d}z_{i_1}}{z_{i_1}}\wedge \dots \wedge \frac{\textrm{d}z_{i_l}}{z_{i_l}}. \end{aligned}$$

So $U^*\textrm{d}U$ acts by  on $\ell ^2(\mathbb {Z}^d)\otimes \bigwedge \nolimits ^*\mathbb {C}^{d}$. Therefore, $\textrm{d}+\textrm{d}^*$ is unitarily equivalent to the operatorby the standard representation of $\mathbb {C}\ell _{d,0}$ in Definition and Lemma 2.3. The generators $\gamma ^j$ are “real” in $\mathbb {C}\ell _{d,0}$, hence maps $\bigwedge \nolimits ^*\mathbb {R}^{d}$ to $\bigwedge \nolimits ^*\mathbb {R}^{d}$. The $j$-th position operator  preserves $\ell ^2(\mathbb {Z}^d)_\mathbb {R}$ because  and $k_j$ is real for any $k\in \mathbb {Z}^d$. Therefore the operator  preserves $\ell ^2(\mathbb {Z}^d)_\mathbb {R}\otimes \bigwedge \nolimits ^*\mathbb {R}^{d}$. $\square$

### Kasparov Theory for Real C*-Algebras

In his seminal work [15], Kasparov introduced a bivariant K-theory for $\mathbb {Z}/2$-graded C*-algebras. Elements in his bivariant K-theory group $\operatorname {KK}(A,B)$ may be viewed as “generalised homomorphisms” from $A$ to $B$. Kasparov’s constructions are general enough to be transferred to the setting of real C*-algebras (cf. [4], Appendix A).

For every pair of $\mathbb {Z}/2$-graded real C*-algebras $A$ and $B$, there is an abelian group (the so-called Kasparov group) $\operatorname {KK}(A,B)$. It generalises the real K-theory of ungraded real C*-algebras (that is, $\operatorname {KO}$-theory) in the following way: if $A=\textrm{C}\ell _{n,0}$ and $B$ is an ungraded real C*-algebra, then there is an isomorphism (cf. ( [15], Section 5, Theorem 7; [3], Lemma 9.1)):

2.7 $$\begin{aligned} \operatorname {KO}_n(B)\simeq \operatorname {KK}(\textrm{C}\ell _{n,0},B). \end{aligned}$$

Given $\mathbb {Z}/2$-graded real C*-algebras $A_1,A_2,B_1,B_2,D$, there is a natural group homomorphism

$$\begin{aligned} \times _D:\operatorname {KK}(A_1,B_1\otimes D)\times \operatorname {KK}(A_2\otimes D,B_2)\rightarrow \operatorname {KK}(A_1\otimes A_2,B_1\otimes B_2) \end{aligned}$$

called the *Kasparov product*, cf. ( [15], Section 4, Theorem 4). We shall need the following formal constructions with the Kasparov product, and refer to [4, 15] for details.

#### Functoriality

Let $f:A\rightarrow B$ and $g:B\rightarrow C$ be *-homomorphisms between real C*-algebras. Then $f$ gives an element $[f]\in \operatorname {KK}(A,B)$ and $[g]\in \operatorname {KK}(B,C)$. Moreover, we have

$$\begin{aligned} [f]\times _B[g]=[g\circ f]\in \operatorname {KK}(A,C). \end{aligned}$$

#### Graded $\operatorname {KO}_{*}(\mathbb {R})$-Module Structure of $\operatorname {KO}_{*}(B)$

Let $A_1=\textrm{C}\ell _{n,0}$, $A_2=\textrm{C}\ell _{m,0}$, $B_1=B_2=D=\mathbb {R}$. Then the Kasparov product gives a group homomorphism

$$\begin{aligned} \times _\mathbb {R}:\underbrace{\operatorname {KK}(\textrm{C}\ell _{n,0},\mathbb {R})}_{\simeq \operatorname {KO}_n(\mathbb {R})}\times \underbrace{\operatorname {KK}(\textrm{C}\ell _{m,0},\mathbb {R})}_{\simeq \operatorname {KO}_m(\mathbb {R})}\rightarrow \underbrace{\operatorname {KK}(\textrm{C}\ell _{m+n,0},\mathbb {R})}_{\simeq \operatorname {KO}_{m+n}(\mathbb {R})}, \end{aligned}$$

where the isomorphisms are given as in (2.7). This turns $\operatorname {KO}_*(\mathbb {R})\mathrel {:=}\bigoplus _{n}\operatorname {KO}_n(\mathbb {R})$ into a graded commutative ring.

Now let $A_1=\textrm{C}\ell _{n,0}$, $A_2=\textrm{C}\ell _{m,0}$, $B_1=D=\mathbb {R}$ and $B_2=B$ be an ungraded real C*-algebra. Then the Kasparov product gives a group homomorphism

$$\begin{aligned} \times _\mathbb {R}:\underbrace{\operatorname {KK}(\textrm{C}\ell _{n,0},\mathbb {R})}_{\simeq \operatorname {KO}_n(\mathbb {R})}\times \underbrace{\operatorname {KK}(\textrm{C}\ell _{m,0},B)}_{\simeq \operatorname {KO}_m(B)}\rightarrow \underbrace{\operatorname {KK}(\textrm{C}\ell _{m+n,0},B)}_{\simeq \operatorname {KO}_{m+n}(B)}, \end{aligned}$$

which turns $\operatorname {KO}_*(B)\mathrel {:=}\bigoplus _{n}\operatorname {KO}_n(B)$ into a graded commutative module over $\operatorname {KO}_*(\mathbb {R})$.

#### Index Pairing

Let $A_1=\textrm{C}\ell _{n,0}$, $A_2=\textrm{C}\ell _{0,d}$, $B_1=B_2=\mathbb {R}$, then the Kasparov product gives a group homomorphism

$$\begin{aligned} \times _D:\underbrace{\operatorname {KK}(\textrm{C}\ell _{n,0},D)}_{\simeq \operatorname {KO}_n(D)}\times \operatorname {KK}(D\otimes \textrm{C}\ell _{0,d},\mathbb {R})\rightarrow \underbrace{\operatorname {KK}(\textrm{C}\ell _{n,d},\mathbb {R})}_{\simeq \operatorname {KO}_{n-d}(\mathbb {R})}. \end{aligned}$$

Thus every element $\alpha \in \operatorname {KK}(D\otimes \textrm{C}\ell _{0,d},\mathbb {R})$ induces a group homomorphism

2.8 $$\begin{aligned} \times _D\alpha :\operatorname {KO}_n(D)\rightarrow \operatorname {KO}_{n-d}(\mathbb {R}). \end{aligned}$$

Moreover, since the Kasparov product is functorial, $\alpha$ actually induces an $\operatorname {KO}_*(\mathbb {R})$-module homomorphism $\operatorname {KO}_*(D)\rightarrow \operatorname {KO}_{*-d}(\mathbb {R})$.

The group homomorphism (2.8) induced by an element $\alpha \in \operatorname {KK}(D\otimes \textrm{C}\ell _{0,d},\mathbb {R})$ is called an *index pairing*. Such an element $\alpha$ can be represented by a real spectral triple.

##### Definition 2.9

Let $A$ be a $\mathbb {Z}/2$-graded real C*-algebra. A real spectral triple $(\mathcal {A},\mathcal {H},D)$ over $A$ consists of:

- A $\mathbb {Z}/2$-graded real Hilbert space $\mathcal {H}$, together with a grading-preserving *-representation $\varphi :A\rightarrow \mathbb {B}(\mathcal {H})$;
- A dense *-subalgebra $$ \mathcal {A}\subseteq A $$;
- An unbounded, self-adjoint odd operator $D:\operatorname {dom}D\subseteq \mathcal {H}\rightarrow \mathcal {H}$;

such that:

- $\varphi (a)(1+D^2)^{-1}$ is compact for all $a\in \mathcal {A}$;
- For every $a\in \mathcal {A}$, $\varphi (a)$ maps $\operatorname {dom}D$ into $\operatorname {dom}D$; and the graded commutator $[D,\varphi (a)]$ extends to a bounded operator on $\mathcal {H}$.

Let $\xi \mathrel {:=}(\mathcal {B},\mathcal {H},D)$ be a real spectral triple over a real C*-algebra $B$, where $B$ is represented on $\mathcal {H}$ via $\varphi :B\rightarrow \mathbb {B}(\mathcal {H})$. Then $\xi$ represents a class $[\xi ]\in \operatorname {KK}(B,\mathbb {R})$.

Let $f:A\rightarrow B$ be a *-homomorphism. Then the *-algebra $\mathcal {A}\mathrel {:=}f^{-1}(\mathcal {B})$ is dense in $A$, and $f^*\xi \mathrel {:=}(\mathcal {A},\mathcal {H},D)$ is a real spectral triple over $A$, where $A$ is represented on $\mathcal {H}$ via $\varphi \circ f:A\rightarrow \mathbb {B}(\mathcal {H})$.

We call $f^*\xi \mathrel {:=}(\mathcal {A},\mathcal {H},D)$ in the above example, the *pullback* spectral triple of $(\mathcal {B},\mathcal {H},D)$ along $f$. Functoriality of Kasparov product states the following:

##### Lemma 2.10

Let $f:A\rightarrow B$ be a *-homomorphism between real C*-algebras. Let $\xi$ be a real spectral triple which represents a class $[\xi ]\in \operatorname {KK}(B\otimes \textrm{C}\ell _{0,d},\mathbb {R})$. Then the pullback spectral triple $f^*\xi$ represents the Kasparov product $[f]\times _B[\xi ]\in \operatorname {KK}(A\otimes \textrm{C}\ell _{0,d},\mathbb {R})$. Moreover, the following diagram commutes: 
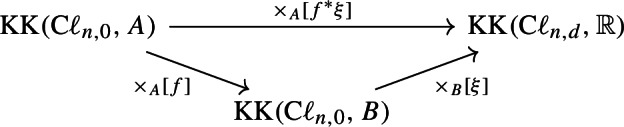
 where all arrows are given by taking Kasparov products.

### Position Spectral Triples

In the following, we focus on a class of spectral triples, which we call *position spectral triples* as they are built from the position operators on the corresponding Hilbert space of a discrete set $\Lambda$.

#### Definition 2.11

Let $\Lambda \subseteq \mathbb {R}^d$ be a countable discrete subset. A *position spectral triple* associated to $\Lambda$ is a real spectral triple of the form2.12where:

- $A$ is a real C*-algebra, which carries a *-representation $\varphi :A\rightarrow \mathbb {B}(\ell ^2(\Lambda )\otimes \mathcal {K})$ for some separable real Hilbert space $\mathcal {K}$; $\mathcal {A}\subseteq A$ is a dense *-subalgebra;
- $A\otimes \textrm{C}\ell _{0,d}$ is represented on $\ell ^2(\Lambda )\otimes \mathcal {K}\otimes \bigwedge \nolimits ^*\mathbb {R}^{d}$ through the tensor product of $\varphi$ and the standard representation of $\textrm{C}\ell _{0,d}$;
is the $j$-th position operator on $\ell ^2(\Lambda )$, given by
- $\gamma _1,\dots ,\gamma _d$ are the generators of $\textrm{C}\ell _{d,0}$, represented on $\bigwedge \nolimits ^*\mathbb {R}^{d}$ via the standard representation.

The condition $\xi _\Lambda$ being a real spectral triple says that:

for all $$ a\in \mathcal {A} $$;
- For every $a\in \mathcal {A}$, $\varphi (a)$ maps  into , and  extends to a bounded operator on $\ell ^2(\Lambda )\otimes \mathcal {K}\otimes \bigwedge \nolimits ^*\mathbb {R}^{d}$.

We note the occurence of an auxiliary separable Hilbert space $\mathcal {K}$. The physical meaning of $\mathcal {K}$ is given in Remark 3.2 for $\Lambda$ a Delone set (cf. Definition 3.1).

The author thanks the anonymous referee for pointing out the following result:

#### Lemma 2.13

Every position spectral triple is *locally compact*. That is, the matrix elementsare compact for all $a\in \mathcal {A}$ and $x,y\in \Lambda$.

#### Proof

By assumption of a real spectral triple, we have thatfor all $a\in \mathcal {A}$. Therefore,is a compact operator on $\mathcal {K}\otimes \bigwedge \nolimits ^*\mathbb {R}^{d}$. This forces $\varphi (a)_{x,y}\in \mathbb {K}(\mathcal {K})$. $\square$

#### The Fundamental Class of $\textrm{C}^*(\mathbb {Z}^d)_\mathbb {R}\otimes \textrm{C}\ell _{0,d}$

The last goal of this section is to construct a position spectral triple $\xi _{\mathbb {Z}^d,N}$ over $\textrm{C}^*(\mathbb {Z}^d)_\mathbb {R}\otimes \mathbb {M}_N(\mathbb {R})\otimes \textrm{C}\ell _{0,d}$, where $\textrm{C}^*(\mathbb {Z}^d)_\mathbb {R}$ is the real group C*-algebra of $\mathbb {Z}^d$. Moreover, we shall show that taking the Kasparov product with the class $[\xi _{\mathbb {Z}^d,N}]\in \operatorname {KK}(\textrm{C}^*(\mathbb {Z}^d)_\mathbb {R}\otimes \textrm{C}\ell _{0,d},\mathbb {R})$ gives a surjective homomorphism

$$\begin{aligned} \operatorname {KK}(\textrm{C}\ell _{d,0},\textrm{C}^*(\mathbb {Z}^d)_\mathbb {R})\rightarrow \operatorname {KK}(\textrm{C}\ell _{d,d},\mathbb {R})\simeq \mathbb {Z}. \end{aligned}$$

Let $\textrm{C}_\textrm{c}(\mathbb {Z}^d)_\mathbb {R}$ be the real convolution algebra of $\mathbb {Z}^d$, whose elements are real-valued, finitely supported functions $f:\mathbb {Z}^d\rightarrow \mathbb {R}$, and carries the following convolution product and involution:

$$\begin{aligned} (f*g)(x)\mathrel {:=}\sum _{x_1+x_2=x}f(x_1)g(x_2),\quad f^*(x)\mathrel {:=}f(-x). \end{aligned}$$

The left multiplication of $\textrm{C}_\textrm{c}(\mathbb {Z}^d)_\mathbb {R}$ on itself extends to a injective *-representation

$$\begin{aligned} \lambda :\textrm{C}_\textrm{c}(\mathbb {Z}^d)_\mathbb {R}\rightarrow \mathbb {B}(\ell ^2(\mathbb {Z}^d,\mathbb {R})). \end{aligned}$$

The closure of $\lambda (\textrm{C}_\textrm{c}(\mathbb {Z}^d)_\mathbb {R})$ in the real C*-algebra $\mathbb {B}(\ell ^2(\mathbb {Z}^d,\mathbb {R}))$ is the real group C*-algebra $\textrm{C}^*(\mathbb {Z}^d)_\mathbb {R}$.

Fix a natural number $N$ and extend $\lambda$ entrywise to $\textrm{C}^*(\mathbb {Z}^d)_\mathbb {R}\otimes \mathbb {M}_N(\mathbb {R})$. This gives a representation

$$\begin{aligned} \lambda \otimes \operatorname {id}_N:\textrm{C}^*(\mathbb {Z}^d)_\mathbb {R}\otimes \mathbb {M}_N(\mathbb {R})\rightarrow \mathbb {B}(\ell ^2(\mathbb {Z}^d,\mathbb {R})\otimes \mathbb {R}^N)\simeq \mathbb {B}(\ell ^2(\mathbb {Z}^d,\mathbb {R}^N)). \end{aligned}$$

To get a position spectral triple over $\mathbb {M}_N(\textrm{C}^*(\mathbb {Z}^d)_\mathbb {R})$, we need another separable real Hilbert space $\mathcal {K}$. Let $\mathbb {R}^N\hookrightarrow \mathcal {K}$ be any isometric embedding. Then it induces a corner embedding $e_N:\mathbb {M}_N(\mathbb {R})\hookrightarrow \mathbb {K}(\mathcal {K})$ and gives an invertible element in $\operatorname {KK}(\mathbb {M}_N(\mathbb {R}),\mathbb {R})$. We represent $\textrm{C}^*(\mathbb {Z}^d)_\mathbb {R}\otimes \mathbb {M}_N(\mathbb {R})$ on $\ell ^2(\mathbb {Z}^d)\otimes \mathcal {K}$ via $\lambda \otimes e_N$. This gives a position spectral triple2.14which represents a class $[\xi _{\mathbb {Z}^d,N}]\in \operatorname {KK}(\mathbb {M}_N(\textrm{C}^*(\mathbb {Z}^d)_\mathbb {R})\otimes \textrm{C}\ell _{0,d},\mathbb {R})$. This is the same class for each $N$ (hence also for $N=1$) because $[e_N]\in \operatorname {KK}(\mathbb {M}_N(\mathbb {R}),\mathbb {R}))$ is invertible.

##### Theorem 2.15

Taking the Kasparov product with $[\xi _{\mathbb {Z}^d,N}]$,

$$\begin{aligned} \times _{\mathbb {M}_N(\textrm{C}^*(\mathbb {Z}^d)_\mathbb {R})}[\xi _{\mathbb {Z}^d,N}]:\operatorname {KK}(\textrm{C}\ell _{d,0},\mathbb {M}_N(\textrm{C}^*(\mathbb {Z}^d)_\mathbb {R}))\rightarrow \operatorname {KK}(\textrm{C}\ell _{d,d},\mathbb {R})\simeq \mathbb {Z}\end{aligned}$$

is a surjective group homomorphism.

##### Proof

The result is well-known, cf. ( [15], Section 5, Theorem 7), whilst we need to appeal to a variant of KK-theory for “real” C*-algebras called KKR-theory (also defined by Kasparov in [15]).

Let $(\mathbb {T}^d,\tau :z\mapsto \overline{z})$ be the “real” $d$-torus. Then Kasparov showed that the “real” spectral triple

2.16 $$\begin{aligned} \left( \textrm{C}(\mathbb {T}^d,\mathbb {C}\ell (\mathbb {T}^d)),\; L^2(\bigwedge \nolimits ^*\textrm{T}^*\mathbb {T}^d),\; \textrm{d}+\textrm{d}^*\right) \end{aligned}$$

represents the KKR-thereotic fundamental class in $\operatorname {KKR}(\textrm{C}(\mathbb {T}^d,\mathbb {C}\ell (\mathbb {T}^d)),\mathbb {C})$. That is, there exists a class $\beta \in \operatorname {KKR}(\mathbb {C},\textrm{C}(\mathbb {T}^d,\mathbb {C}\ell (\mathbb {T}^d)))$ (“the Bott class”) such that

$$\begin{aligned} \beta \times _{\textrm{C}(\mathbb {T}^d,\mathbb {C}\ell (\mathbb {T}^d))}\alpha =1\in \operatorname {KKR}(\mathbb {C},\mathbb {C}). \end{aligned}$$

Now we pass from KKR to KK-theory of real C*-algebras. By Lemma 2.4, the Fourier transform (2.5) maps $\textrm{C}(\mathbb {T}^d,\mathbb {C}\ell (\mathbb {T}^d))$ to $\textrm{C}^*(\mathbb {Z}^d)\otimes \mathbb {C}\ell _{0,d}$, where $\textrm{C}^*(\mathbb {Z}^d)$ carries the real structure $\mathfrak {r}(f)(x)=\overline{f(x)}$; and the Hodge–de Rham operator $\textrm{d}+\textrm{d}^*$ to the position operator , which is a “real” operator.

There is a natural isomorphism $\operatorname {KKR}(A,B)\simeq \operatorname {KK}(A^{\mathfrak {r}_A},B^{\mathfrak {r}_B})$ (cf. [7], Section 2). Under this isomorphism, we find that the Hodge–de Rham spectral triple (2.16), after composing with a rank-one corner embedding $\mathbb {R}\hookrightarrow \mathbb {K}(\mathcal {K})$, is mapped to the position spectral triple $\xi _{\mathbb {Z}^d,1}$. Hence its Kasparov product with the Bott class in $\operatorname {KK}(\mathbb {R},\textrm{C}^*(\mathbb {Z}^d)_\mathbb {R}\otimes \textrm{C}\ell _{0,d})$ equals $1\in \mathbb {Z}\simeq \operatorname {KK}(\textrm{C}\ell _{d,d},\mathbb {R})$. $\square$

##### Remark 2.17

The KKR-class of the Hodge–de Rham spectral triple (2.16) is also called the Dirac element (cf. ( [16], Definition–Lemma 4.2)). It is the KK-theoretic analogue of the fundamental class in cohomology. We refer to ( [21], Section 4.3) for the notion of fundamental classes in Kasparov theory and noncommutative geometry.

## Models of Aperiodic Topological Insulators

### Convention

From now on, all Hilbert spaces and C*-algebras are assumed to be real. All C*-algebras excepted for the Clifford algebras (and their tensor products with other C*-algebras) are assumed to be ungraded.

In this section, we shall describe the groupoid model (cf. Section 3.1) and Roe C*-algebra model (cf. Section 3.2) of topological insulators on aperiodic lattices. A short discussion on the *uniform* Roe C*-algebras is given in Section 3.2.3.

We assume that the aperiodic lattice is described by a Delone set $\Lambda \subseteq \mathbb {R}^d$, i.e. a uniformly discrete and relatively dense subset of $\mathbb {R}^d$, defined as follows.

#### Definition 3.1

Let $\Lambda \subseteq \mathbb {R}^d$ be a discrete infinite set. Fix $0<r<R$. Then $\Lambda$ is called

- $r$-*uniformly discrete*, if $\#(\operatorname {B}(x,r)\cap \Lambda )\le 1$ for all $x\in \mathbb {R}^d$;
- $R$-*relatively dense*, if $\#(\operatorname {B}(x,R)\cap \Lambda )\ge 1$ for all $x\in \mathbb {R}^d$;
- $(r,R)$-*Delone*, if $\Lambda$ is both $r$-uniformly discrete and $R$-relatively dense.

Denote the collection of $(r,R)$-Delone sets in $\mathbb {R}^d$ by $\operatorname {Del}_{(r,R)}(\mathbb {R}^d)$.

#### Remark 3.2

**Fig. 1:**
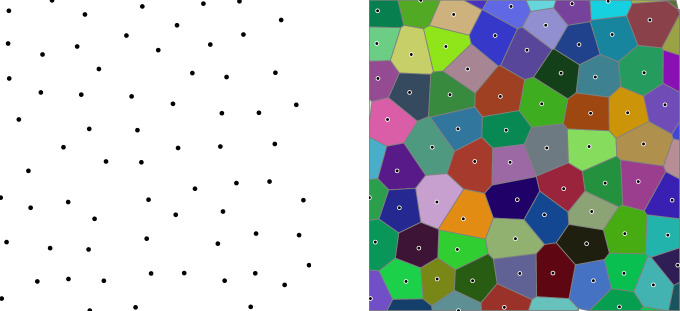
Voronoi tiling associated to a Delone set

Given a Delone set $\Lambda$, equipped with the subspace metric $d(x,y)\mathrel {:=}\left|x-y\right|$. The Voronoi tiling (cf. Figure 1) decomposes $\mathbb {R}^d$ into tiles lablled by $x\in \Lambda$, and thus decomposes the Hilbert space $L^2(\mathbb {R}^d)$ into a direct sum:

3.3 $$\begin{aligned} L^2(\mathbb {R}^d)\simeq \bigoplus _{x\in \Lambda }L^2(T_x), \end{aligned}$$

where $T_x$ is (interior of) the tile associated to the point $x\in \Lambda$. If $\Lambda$ is a periodic lattice, then all $T_x$’s are translated copies of the fundamental domain of $\Lambda$, where $\Lambda$ is identified with the discrete, cocompact subgroup of $\mathbb {R}^d$ generated by translations by vectors $x\in \Lambda$. In this sense, we may regard $T_x$’s as a generalised version of “fundamental domains” of the dynamics on the aperiodic lattice $\Lambda$.

We may further identify all $L^2(T_x)$’s with a fixed, separable Hilbert space $\mathcal {K}$. Thus an operator $T\in \mathbb {B}(L^2(\mathbb {R}^d))\simeq \mathbb {B}(\ell ^2(\Lambda )\otimes \mathcal {K})$ can be described by its matrix elements $(T_{x,y})_{x,y\in \Lambda }$, where

### Groupoid C*-Algebra of a Delone Set

In the groupoid approach, a Delone set $\Lambda$ is considered as a point in the space of infinite, discrete subsets in $\mathbb {R}^d$. Its dynamics is given by translation of the point set by vectors in $\mathbb {R}^d$. More precisely, we identify a Delone set $\Lambda$ with its corresponding pure point measure $\sum _{x\in \Lambda }\delta _x$ on $\mathbb {R}^d$, thus identify the space of all $(r,R)$-Delone sets as a subspace of $\mathcal {M}(\mathbb {R}^d)=\textrm{C}_\textrm{c}(\mathbb {R}^d)'$, the space of all measures on $\mathbb {R}^d$, equipped with the weak*-topology. Following [6], we write:

- $\mathcal {M}(\mathbb {R}^d)$ for the space of all measures on $\mathbb {R}^d$;
- $\operatorname {QD}(\mathbb {R}^d)$ for the set of all pure point measures $\nu$ on $\mathbb {R}^d$ such that $\nu (\{x\})\in \mathbb {N}$ for all $x\in \mathbb {R}^d$;
- $\operatorname {UD}_r(\mathbb {R}^d)$ for the subset of $\operatorname {QD}(\mathbb {R}^d)$ such that $\nu (\operatorname {B}(x;r))\le 1$ for all $x\in \mathbb {R}^d$.

In the context below, we shall not distinguish between a discrete set and its corresponding pure point measure.

#### Proposition 3.4

([6], Theorem 1.5, Section 2.1) Using the notation above and fix $0<r<R$, we have the following:

1. there are inclusions of *closed* sets
  $$\begin{aligned} \operatorname {Del}_{(r,R)}(\mathbb {R}^d)\subsetneqq \operatorname {UD}_r(\mathbb {R}^d)\subsetneqq \operatorname {QD}(\mathbb {R}^d)\subsetneqq \mathcal {M}(\mathbb {R}^d); \end{aligned}$$
2. the space $\operatorname {QD}(\mathbb {R}^d)$ is complete and metrisable;
3. the space $\operatorname {UD}_r(\mathbb {R}^d)$ is compact.

As a corollary of (1)–(3), $\operatorname {Del}_{(r,R)}(\mathbb {R}^d)$ is a compact metrisable space.

The space $\operatorname {Del}_{(r,R)}(\mathbb {R}^d)$ carries an action of $\mathbb {R}^d$ by translation. That is, given $\Lambda \in \operatorname {Del}_{(r,R)}(\mathbb {R}^d)$, viewed as a discrete point set in $\mathbb {R}^d$; and given $a\in \mathbb {R}^d$, then $\Lambda +a$ is the discrete point set consisting of $x+a$ for all $x\in \Lambda$. This coincides with the $\mathbb {R}^d$-action on the space of measures.

From now on we shall fix a Delone set $\Lambda$. Let $\Omega _\Lambda$ be the *closure* of the orbit of $\Lambda$ under the $\mathbb {R}^d$-action. Then $\Omega _\Lambda$ is a disjoint union of orbits, hence closed under the $\mathbb {R}^d$-action. This allows us to restrict the topological dynamical system $\operatorname {Del}_{(r,R)}(\mathbb {R}^d)\curvearrowleft \mathbb {R}^d$ to the smaller space $\Omega _\Lambda$ and construct the *action groupoid* $\Omega _\Lambda \rtimes \mathbb {R}^d$. The elements are of the form $(\omega ,a)\in \Omega _{\Lambda }\times \mathbb {R}^d$ and the structure maps are given by

$$\begin{aligned} \qquad r(\omega ,a)&=\omega , \qquad \qquad \qquad \qquad \qquad s(\omega ,a)=\omega -a,\\ (\omega ,a)\cdot (\omega -a,b)&=(\omega ,a+b), \quad \!\qquad \qquad (\omega ,a)^{-1}=(\omega -a,-a). \end{aligned}$$

An *abstract transversal* of a topological groupoid $\mathcal {G}$ is a closed subset $X\subseteq \mathcal {G}^{0}$ such that $X$ meets every $\mathcal {G}$-orbit of $\mathcal {G}^{0}$; and the restrictions of the range and source maps to $\mathcal {G}_X\mathrel {:=}s^{-1}(X)$ are open, cf. ( [9], Definition 2.11, [24], Example 2.7). Retricting a topological groupoid $\mathcal {G}$ to an abstract transversal $X$ yields a Morita equivalent groupoid $\mathcal {G}_X^X\mathrel {:=}s^{-1}(X)\cap r^{-1}(X)$. The Morita equivalence is implemented by the space $\mathcal {G}_X$.

#### Definition and Lemma 3.5

([9], Proposition 2.14) The action groupoid $\Omega _\Lambda \rtimes \mathbb {R}^d$ admits the following abstract transversal

$$\begin{aligned} \Omega _0\mathrel {:=}\lbrace \omega \in \Omega _{\Lambda }\mid 0\in \omega \rbrace . \end{aligned}$$

Define the groupoid $\mathcal {G}_\Lambda$ as the restriction of $\Omega _{\Lambda }\rtimes \mathbb {R}^d$ to the transversal $\Omega _0$. Then $\mathcal {G}_\Lambda$ is an étale groupoid, which is Morita equivalent to $\Omega _{\Lambda }\rtimes \mathbb {R}^d$.

#### The Regular Representation

The reduced real C*-algebra of an étale groupoid $\mathcal {G}$ is the completion of the real groupoid convolution algebra $\textrm{C}_\textrm{c}(\mathcal {G})$ under the regular representation (cf. ( [18])), which we recall here. The real groupoid convolution algebra $\textrm{C}_\textrm{c}(\mathcal {G})$ consists of real-valued, compactly supported continuous functions $f:\mathcal {G}\rightarrow \mathbb {R}$, equipped with the convolution product and involution

$$\begin{aligned} f*g(\gamma )\mathrel {:=}\sum _{\gamma _1\gamma _2=\gamma }f(\gamma _1)g(\gamma _2),\quad f^*(\gamma )\mathrel {:=}f(\gamma ^{-1}). \end{aligned}$$

The space $\textrm{C}_\textrm{c}(\mathcal {G})$ is a pre-Hilbert module over $\textrm{C}_0(\mathcal {G}^0)$, with right $\textrm{C}_0(\mathcal {G}^0)$-module structure and inner product (cf. [9], Section 1.3)

$$\begin{aligned} (f\cdot \varphi )(\gamma )&\mathrel {:=}f(\gamma )\varphi (s(\gamma )),\\ \left\langle f,g\right\rangle (x)&\mathrel {:=}(f^**g)\big |_{\mathcal {G}^0}(x)=\sum _{\gamma \in r^{-1}(x)}f(\gamma )g(\gamma ), \end{aligned}$$

for $\gamma \in \mathcal {G}$, $x\in \mathcal {G}^0$, $\varphi \in \textrm{C}_0(\mathcal {G}^0)$ and $f,g\in \textrm{C}_\textrm{c}(\mathcal {G})$. Write $L^2(\mathcal {G})$ for the Hilbert C*-module completion of $\textrm{C}_\textrm{c}(\mathcal {G})$. Then the left multiplication

$$\begin{aligned} \pi (f):\textrm{C}_\textrm{c}(\mathcal {G})\rightarrow \textrm{C}_\textrm{c}(\mathcal {G}),\quad g\mapsto f*g \end{aligned}$$

extends to a *-representation

$$\begin{aligned} \pi :\textrm{C}_\textrm{c}(\mathcal {G})\rightarrow \mathbb {B}_{\textrm{C}_0(\mathcal {G}^0)}(L^2(\mathcal {G})), \quad f\mapsto \pi (f), \end{aligned}$$

representing $\textrm{C}_\textrm{c}(\mathcal {G})$ by adjointable operators on Hilbert $\textrm{C}_0(\mathcal {G}^0)$-module $L^2(\mathcal {G})$.

##### Definition 3.6

The reduced groupoid C*-algebra $\textrm{C}^*(\mathcal {G})$ of an étale groupoid $\mathcal {G}$ is the completion of $\textrm{C}_\textrm{c}(\mathcal {G})$ in the norm $f\mapsto \left\Vert \pi (f)\right\Vert$.

Let $x\in \mathcal {G}^0$. Then the evaluation *-homomorphism

$$\begin{aligned} \operatorname {ev}_x:\textrm{C}_0(\mathcal {G}^0)\rightarrow \mathbb {R},\quad \operatorname {ev}_x(\varphi )\mathrel {:=}\varphi (x) \end{aligned}$$

induces a *-homomorphism

$$\begin{aligned} (\operatorname {ev}_x)_*:\mathbb {B}_{\textrm{C}_0(\mathcal {G}^0)}(L^2(\mathcal {G}))\rightarrow \mathbb {B}(L^2(\mathcal {G})\otimes _{\operatorname {ev}_x}\mathbb {R}),\quad T\mapsto T\otimes \operatorname {id}. \end{aligned}$$

Denote by $\mathcal {G}_x$ the source fibre of $\mathcal {G}$ at $x$. The Hilbert space

$$\begin{aligned} \mathcal {H}_x\mathrel {:=}L^2(\mathcal {G})\otimes _{\operatorname {ev}_x}\mathbb {R}\end{aligned}$$

is isomorphic to $\ell ^2(\mathcal {G}_x)$, sending $f\otimes t$ to the restriction of $t\cdot f$ to $\mathcal {G}_x$. Thus $\pi _x\mathrel {:=}(\operatorname {ev}_x)_*\circ \pi$ gives a representation of $\textrm{C}^*(\mathcal {G})$ on $\mathcal {H}_x$. We call it the *localised* representation (of the regular representation $\pi$) at $x\in \mathcal {G}^0$; and call $\mathcal {H}_x$ the *localised* Hilbert space at $x\in \mathcal {G}^0$.

Now we let $\mathcal {G}$ be the groupoid of Delone sets $\mathcal {G}_\Lambda$. By definition, its regular representation is defined on the Hilbert $\textrm{C}(\Omega _0)$-module $L^2(\mathcal {G}_\Lambda )_{\textrm{C}(\Omega _0)}$. This is a Hilbert module over a unital, commutative C*-algebra, and hence equivalent to a continuous field of Hilbert spaces over $\Omega _0$. The source fibre of $\mathcal {G}_\Lambda$ at $\omega \in \Omega _0$ is given by

$$\begin{aligned} \left\rbrace (\omega -x,-x)\;\big |\;0\in \omega -x\right\lbrace =\left\rbrace (\omega -x,-x)\;\big |\;x\in \omega \right\lbrace , \end{aligned}$$

which is in bijection with the Delone set $\omega$ via $(\omega -x,-x)\mapsto x$. Thus the localised Hilbert space $\mathcal {H}_\omega$ is unitarily isomorphic to $\ell ^2(\omega )$ via (cf. [9], Section 4.1):

3.7 $$\begin{aligned} \begin{aligned} \rho _\omega :\mathcal {H}_\omega \xrightarrow {\sim }\ell ^2(s^{-1}(\omega )),&\qquad \rho _\omega (f\otimes t)(x)\mathrel {:=}tf(\omega -x,-x).\\ \rho _\omega ^{-1}:\ell ^2(s^{-1}(\omega ))\xrightarrow {\sim }\mathcal {H}_\omega ,&\qquad \rho _\omega ^{-1}(\left|x\right\rangle )\mathrel {:=}\left|\omega -x,-x\right\rangle , \end{aligned} \end{aligned}$$

where $\left|\omega -x,-x\right\rangle$ is the equivalence class in $\mathcal {H}_\omega$ of any continuous function $f$ on $\mathcal {G}$, such that $\operatorname {supp}f\cap s^{-1}(\omega )=\{(\omega -x,-x)\}$ and $f(\omega -x,-x)=1$.

##### Lemma 3.8

([9], Section 4.1) The localised representation $\pi _\omega :\textrm{C}^*(\mathcal {G}_\Lambda )\rightarrow \mathbb {B}(\ell ^2(\omega ))$ is given by the formula

3.9 $$\begin{aligned} (\pi _\omega (f)\psi )(x)\mathrel {:=}\sum _{y\in \omega }f(\omega -x,y-x)\psi (y) \end{aligned}$$

for $f\in \textrm{C}^*(\mathcal {G}_\Lambda )$, $\psi \in \ell ^2(\omega )$ and $x\in \omega$. Therefore,

When we consider systems with a finite number of internal degrees of freedom inside every lattice site, e.g. spins of electrons, then we must replace the observable C*-algebra by its matrix algebra $\mathbb {M}_N\textrm{C}^*(\mathcal {G}_{\Lambda }) =\textrm{C}^*(\mathcal {G}_\Lambda )\otimes \mathbb {M}_N(\mathbb {R})$. In the same way as Section 2.3, we extend the regular representation of $\textrm{C}^*(\mathcal {G}_\Lambda )$ to a representation

$$\begin{aligned} \pi _\omega ^N\mathrel {:=}\pi _\omega \otimes e_N,\quad \textrm{C}^*(\mathcal {G}_\Lambda )\otimes \mathbb {M}_N(\mathbb {R})\rightarrow \mathbb {B}(\ell ^2(\omega )\otimes \mathcal {K}), \end{aligned}$$

where $\mathcal {K}$ is a separable Hilbert space, and $e_N:\mathbb {M}_N(\mathbb {R})\hookrightarrow \mathbb {K}(\mathcal {K})$ is any rank-$N$ corner embedding induced by an isometry $\mathbb {R}^N\hookrightarrow \mathcal {K}$. We may describe the representation $\pi _\omega ^N$ by an infinite matrix indexed by $x,y\in \omega$, with matrix elements3.10for all $f\in \textrm{C}^*(\mathcal {G}_\Lambda )$ and $S\in \mathbb {M}_N(\mathbb {R})$.

#### Position Spectral Triple Over the Groupoid C*-Algebra

In order to construct a spectral triple over $\textrm{C}^*(\mathcal {G}_\Lambda )$ or $\mathbb {M}_N\textrm{C}^*(\mathcal {G}_\Lambda )$, one way is to first construct an unbounded Kasparov $\textrm{C}^*(\mathcal {G}_\Lambda )$-$\textrm{C}(\Omega _0)$–module, then take its (unbounded) Kasparov product with a *-homomorphism $\textrm{C}(\Omega _0)\rightarrow \mathbb {R}$. Such an unbounded Kasparov module was given in ( [9], Section 2.3.1) using the “position” operators on the Hilbert $\textrm{C}(\Omega _0)$-module $L^2(\mathcal {G}_\Lambda )$:

3.11 $$\begin{aligned}  _{d}{\lambda }_{\Omega _0}\mathrel {:=}\left( \textrm{C}_\textrm{c}(\mathcal {G}_\Lambda )\otimes \textrm{C}\ell _{0,d},\quad L^2(\mathcal {G}_\Lambda )_{\textrm{C}(\Omega _0)}\otimes \bigwedge \nolimits ^*\mathbb {R}^{d},\quad \sum _{i=1}^dX_j\otimes \gamma ^j\right) , \end{aligned}$$

where

$$\begin{aligned} c_j:\mathcal {G}_\Lambda \rightarrow \mathbb {R},\quad c_j(\omega ,x)\mathrel {:=}x_j; \qquad X_jf(\omega ,x)\mathrel {:=}c_j(\omega ,x)f(\omega ,x). \end{aligned}$$

The map $c_j:\mathcal {G}_\Lambda \rightarrow \mathbb {R}$ is an *exact* groupoid cocycle in the sense of ( [22], Definition 4.1.2). It follows from the general construction in ( [22], Theorem 3.2.2) that $_{d}{\lambda }_{\Omega _0}$ is an unbounded Kasparov module. Following [9], we call $_{d}{\lambda }_{\Omega _0}$ the *bulk cycle*.

Now we construct the position spectral triple by localising $_{d}{\lambda }_{\Omega _0}$ at any $\omega \in \Omega _0$. As explained in the paragraph after Definition 3.1, the physically relevant Hilbert space is $\ell ^2(\omega )\otimes \mathcal {K}$ instead of $\ell ^2(\omega )$ or $\ell ^2(\omega )\otimes \mathbb {R}^N$. This is fixed by taking any isometry $\mathbb {R}^N\hookrightarrow \mathcal {K}$ and its induced corner embedding $e_N:\mathbb {M}_N(\mathbb {R})\hookrightarrow \mathbb {K}(\mathcal {K})$.

##### Theorem 3.12

Let $\omega \in \Omega _0$ and $\mathcal {K}$ be a Hilbert space. The following data3.13is a position spectral triple. It represents a class in

$$\begin{aligned} \operatorname {KK}(\mathbb {M}_N\textrm{C}^*(\mathcal {G}_\Lambda )\otimes \textrm{C}\ell _{0,d},\mathbb {R}) \simeq \operatorname {KK}(\textrm{C}^*(\mathcal {G}_\Lambda )\otimes \textrm{C}\ell _{0,d},\mathbb {R}), \end{aligned}$$

which is the Kasparov product of the following (unbounded) Kasparov modules and *-homomorphisms:

1. the bulk cycle $_{d}{\lambda }_{\Omega _0}$, which gives a class in $\operatorname {KK}(\textrm{C}^*(\mathcal {G}_\Lambda )\otimes \textrm{C}\ell _{0,d},\textrm{C}(\Omega _0))$;
2. the evaluation *-homomorphism $\operatorname {ev}_\omega :\textrm{C}(\Omega _0)\rightarrow \mathbb {R}$, which gives a class in $\operatorname {KK}(\textrm{C}(\Omega _0),\mathbb {R})$;
3. the corner embedding $e_N:\mathbb {M}_N(\mathbb {R})\hookrightarrow \mathbb {K}(\mathcal {K})$, which gives an invertible element in $\operatorname {KK}(\mathbb {M}_N(\mathbb {R}),\mathbb {R})$.

##### Proof

The composition of (1)–(3) is easy to compute because either (2) or (3) can be described by a Hilbert module with zero operator. The tensor product of these Hilbert modules

$$\begin{aligned} \left( L^2(\mathcal {G}_\Lambda )\otimes _{\operatorname {ev}_\omega }\mathbb {R}\otimes \mathcal {K}\right) \otimes \bigwedge \nolimits ^*\mathbb {R}^{d} \end{aligned}$$

is isomorphic to $\ell ^2(\omega )\otimes \mathcal {K}\otimes \bigwedge \nolimits ^*\mathbb {R}^{d}$ via the localised representation at $\omega$. Thus it suffices to check that $c_j$ is mapped to  under the composition and that (3.13) is a spectral triple. By (3.7), the operator $X_j\otimes \operatorname {id}_{\mathcal {K}}\otimes \operatorname {id}_{\bigwedge \nolimits ^*\mathbb {R}^{d}}$ acts on $\ell ^2(\omega )\otimes \mathcal {K}\otimes \bigwedge \nolimits ^*\mathbb {R}^{d}$ sends $\left|x\right\rangle \otimes v\otimes w$ to $x_j\left|x\right\rangle \otimes v\otimes w$, where $x\in \omega$ is considered an element in $\mathbb {R}^d$. It is a spectral triple by ( [9], Proposition 4.1). Thus (3.13) is a position spectral triple. $\square$

### Roe C*-Algebra of a Delone Set

The coarse-geometric approach uses the Roe C*-algebra $\textrm{C}^*_\textrm{Roe}(\Lambda )$ of the Delone set $\Lambda$. It consists of operators on $\ell ^2(\Lambda ,\mathcal {K})$ which are locally compact and can be approximated by controlled operators. In this setting, we treat a Delone set $\Lambda \subseteq \mathbb {R}^d$ as discrete metric space, equipped with the subspace topology from $\mathbb {R}^d$.

#### Definition 3.14

An *ample* $X$-*module* or *ample module over* $X$ is given by a pair $(X,\mathcal {H}_X)$, where $X$ is a proper metric space, and $\mathcal {H}_X$ is a Hilbert space that carries a non-degenerate representation $\varrho :\textrm{C}_0(X)\rightarrow \mathbb {B}(\mathcal {H}_X)$, which is *ample* in the sense that

$\varrho (f)\in \mathbb {K}(\mathcal {H}_X)$   if and only if   $f=0$.

#### Example 3.15

([29], Example 4.1.2–4.1.4) Let $X$ be a Riemannian manifold with volume form $\mu$. Assume that every connected component of $X$ has dimension greater equal than 1, e.g. $X=\mathbb {R}^d$ for $d\ge 1$ with the Lebesgue measure. Then pointwise multiplication gives an ample $X$-module $\mathcal {H}_X\mathrel {:=}L^2(X,\mu )$.

If $X$ is a discrete metric space with the counting measure, then the representation $\textrm{C}_0(X)\rightarrow \mathbb {B}(\ell ^2(X))$ by multiplication is no longer ample. In such cases, we replace $\ell ^2(X)$ by by $\mathcal {H}_X\mathrel {:=}\ell ^2(X,\mathcal {K})=\ell ^2(X)\otimes \mathcal {K}$, where $\mathcal {K}$ is any separable Hilbert space, and the representation maps $f\in \textrm{C}_0(X)$ to the tensor product of pointwise multiplication with $f$ with the identity operator on $\mathcal {K}$. Then $\mathcal {H}_X$ is an ample $X$-module.

We call $\mathcal {H}_X$ in the above cases, the *standard* ample $X$-module.

#### Definition 3.16

([29], Definition 4.1.7 and 5.1.1) Let $(X,\mathcal {H}_X)$ and $(Y,\mathcal {H}_Y)$ be ample modules, carrying ample representations $\varrho _X:\textrm{C}_0(X)\rightarrow \mathbb {B}(\mathcal {H}_X)$ and $\varrho _Y:\textrm{C}_0(Y)\rightarrow \mathbb {B}(\mathcal {H}_Y)$.

- The *support* of an operator $T\in \mathbb {B}(\mathcal {H}_X,\mathcal {H}_Y)$, denoted by $\operatorname {supp}(T)$, is the collection of all points $(y,x)\in Y\times X$, such that $\varrho _Y(\chi _V)T\varrho _X(\chi _U)\ne 0$ holds for all open neighbourhood $U$ of $x$ and $V$ of $y$.
- A subset $\mathcal {E}\subseteq X\times X$ is called *controlled*, if
  $$\begin{aligned} \sup \left\rbrace \textrm{d}_X(x_1,x_2)\;\big |\;(x_1,x_2)\in \mathcal {E}\right\lbrace <+\infty . \end{aligned}$$
- An operator $T\in \mathbb {B}(\mathcal {H}_X)$ is called *locally compact*, if for any $f\in \textrm{C}_\textrm{c}(X)$, $T\varrho (f)$ and $\varrho (f)T$ are compact operators.
- An operator $T\in \mathbb {B}(\mathcal {H}_X)$ is called *controlled*, if $\operatorname {supp}(T)$ is controlled.

#### Definition 3.17

([29], Definition 5.1.4) Let $(X,\mathcal {H}_X )$ be an ample module. The Roe C*-algebra $\textrm{C}^*_\textrm{Roe}(X,\mathcal {H}_X)$ is the C*-algebra generated by all *locally compact*, *controlled* operators on $\mathcal {H}_X$.

#### Example 3.18

([12], Example 1) Let $\Lambda$ be a discrete metric space and $\mathcal {H}_\Lambda =\ell ^2(\Lambda ,\mathcal {K})$ be the standard ample $\Lambda$-module. Describe an operator $T\in \mathbb {B}(\mathcal {H}_\Lambda )$ by an infinite matrix $(T_{x,y})_{x,y\in \Lambda }$ whereThen the support of $T$ is given by

$$\begin{aligned} \operatorname {supp}(T)=\left\rbrace (x,y)\in X\times X\;\big |\;T_{x,y}\ne 0\right\lbrace . \end{aligned}$$

The operator $T$ is locally compact, if and only if $T_{x,y}\in \mathbb {K}(\mathcal {K})$ for all $x,y\in \Lambda$; controlled, if and only if there exists $R>0$, such that $T_{x,y}=0$ whenever $\textrm{d}(x,y)>R$.

A *-homomorphism between Roe C*-algebras can be induced by a certain (not necessarily continuous) map between their underlying spaces. Such maps are called *coarse*. Their induced *-homomorphism are constructed using *covering isometries*.

#### Definition 3.19

([29], Definition 5.1.10) Let $(X,\textrm{d}_X)$ and $(Y,\textrm{d}_Y)$ be proper metric spaces. A map $f:X\rightarrow Y$ is called *coarse*, if the following holds:

1. The *expansion function* $\omega _f:[0,\infty )\rightarrow [0,\infty )$, given by the formula
  $$\begin{aligned} \omega _f(r)\mathrel {:=}\sup \left\rbrace \textrm{d}_Y(f(x_1),f(x_2)) \;\big |\;\textrm{d}_X(x_1,x_2)\le r\right\lbrace \end{aligned}$$
  satisfies $\omega _f(r)<+\infty$ for all $r\ge 0$.
2. The map $f$ is proper, i.e. $f^{-1}(K)\subseteq X$ is pre-compact for any compact set $K\subseteq Y$.

Two coarse maps $f,g:X\rightrightarrows Y$ are *close*, if there exists $c\ge 0$ such that for all $x\in X$, $d_Y(f(x),g(x))\le c$.

#### Definition and Lemma 3.20

([29], Definition 5.1.11, Lemma 5.1.12) Let $(X,\mathcal {H}_X)$ and $(Y,\mathcal {H}_Y)$ be ample modules and let $f:X\rightarrow Y$ be a coarse map. A covering isometry for $f$ is an isometry $V:\mathcal {H}_X\rightarrow \mathcal {H}_Y$ such that there exists $t\ge 0$ such that $\textrm{d}(y,f(x))<t$ whenever $(y,x)\in \operatorname {supp}(V)$.

The *-homomorphism

$$\begin{aligned} \operatorname {Ad}_V:\mathbb {B}(\mathcal {H}_X)\rightarrow \mathbb {B}(\mathcal {H}_Y),\qquad T\mapsto VTV^* \end{aligned}$$

restricts to a *-homomorphism $\textrm{C}^*_\textrm{Roe}(X,\mathcal {H}_X)\rightarrow \textrm{C}^*_\textrm{Roe}(Y,\mathcal {H}_Y)$. Its induced maps

$$\begin{aligned} f_*:\operatorname {KK}(\textrm{C}\ell _{n,0},\textrm{C}^*_\textrm{Roe}(X,\mathcal {H}_X))\rightarrow \operatorname {KK}(\textrm{C}\ell _{n,0},\textrm{C}^*_\textrm{Roe}(Y,\mathcal {H}_Y)) \end{aligned}$$

depends only on $f$ and not on the choice of $V$.

The *coarse category* consists of proper metric spaces as objects, and equivalence classes of coarse maps as arrows, where two coarse maps are equivalent if and only if they are close. A *coarse equivalence* is an isomorphism in the coarse category. If $X\subseteq Y$ is a metric subspace that is coarsely equivalent to $Y$, then we also say that $X$ is *coarsely dense* in $Y$. In particular, any Delone set $\Lambda$ of $\mathbb {R}^d$ is coarsely dense in $\mathbb {R}^d$.

#### Proposition 3.21

([29], Proposition 4.3.5) Let $(X,\mathcal {H}_X)$ and $(Y,\mathcal {H}_Y)$ be ample modules and $f:X\rightarrow Y$ be a coarse equivalence, then there exists a covering isometry $V:\mathcal {H}_X\rightarrow \mathcal {H}_Y$ that is a unitary equivalence.

As a special case, we have the following:

#### Proposition 3.22

Let $Y$ be a discrete metric space, and $X\subseteq Y$ be a coarsely dense subset. Let $\mathcal {H}_X\mathrel {:=}\ell ^2(X,\mathcal {K})$ and $\mathcal {H}_Y\mathrel {:=}\ell ^2(Y,\mathcal {K})$ be their standard ample modules. Then the operator $V:\mathcal {H}_X\rightarrow \mathcal {H}_Y$, $V\left|x\right\rangle \mathrel {:=}\left|x\right\rangle$ is a covering isometry for the isometric embedding $\iota :X\hookrightarrow Y$ and induce isomorphisms

$$\begin{aligned} \iota _*:\operatorname {KK}(\textrm{C}\ell _{n,0},\textrm{C}^*_\textrm{Roe}(X,\mathcal {H}_X)) \rightarrow \operatorname {KK}(\textrm{C}\ell _{n,0},\textrm{C}^*_\textrm{Roe}(Y,\mathcal {H}_Y)). \end{aligned}$$

#### Proof

The support of $V$ isSo $\textrm{d}(y,\iota (x))=0$ for all $(y,x)\in \operatorname {supp}(V)$. Therefore $V$ is an covering isometry for $\iota$, and $\operatorname {Ad}_V$ induces a *-homomorphism $\iota _*:\textrm{C}^*_\textrm{Roe}(X,\mathcal {H}_X)\rightarrow \textrm{C}^*_\textrm{Roe}(Y,\mathcal {H}_Y)$. Since $\iota$ is a coarse equivalence, there exists a covering isometry $V':\mathcal {H}_X\rightarrow \mathcal {H}_Y$ that is a unitary equivalence. Hence, $\operatorname {Ad}_{V'}$ induces an isomorphism

$$\begin{aligned} \operatorname {KK}(\textrm{C}\ell _{n,0},\textrm{C}^*_\textrm{Roe}(X,\mathcal {H}_X))\rightarrow \operatorname {KK}(\textrm{C}\ell _{n,0},\textrm{C}^*_\textrm{Roe}(Y,\mathcal {H}_Y)). \end{aligned}$$

By Definition and Lemma 3.20, the induced maps of $\operatorname {Ad}_V$ coincides with that of $\operatorname {Ad}_{V'}$, hence an isomorphism as well. $\square$

#### Remark 3.23

By Propositions 3.21 and 3.22, given two Delone set $\Lambda _1,\Lambda _2\subseteq \mathbb {R}^d$ and ample modules $\mathcal {H}_{\Lambda _1},\mathcal {H}_{\Lambda _2}$, then there exists an isomorphism $\textrm{C}^*_\textrm{Roe}(\Lambda _1,\mathcal {H}_{\Lambda _1})\simeq \textrm{C}^*_\textrm{Roe}(\Lambda _2,\mathcal {H}_{\Lambda _2})$. More generally, all of them are isomorphic to the Roe C*-algebra of $\mathbb {R}^d$ defined by its standard ample module, cf. ( [12], Theorem 2). However, we note that the inclusion $\iota :\Lambda \hookrightarrow \mathbb {R}^d$ does *not* induce an isomorphism (via a covering isometry) between the corresponding Roe C*-algebras $\textrm{C}^*_\textrm{Roe}(\Lambda ,\mathcal {H}_\Lambda )$ and $\textrm{C}^*_\textrm{Roe}(\mathbb {R}^d,\mathcal {H}_{\mathbb {R}^d})$ defined using their standard ample modules. An isomorphism between the Roe C*-algebras of coarsely equivalent spaces $X$ and $Y$ was constructed in ( [12], Theorem 3). However, these spaces carry different ample modules, involving an inverse of the coarsely dense embedding $\iota :X\rightarrow X\coprod Y$ in the coarse category.

As opposed to the groupoid C*-algebra $\textrm{C}^*(\mathcal {G}_\Lambda )$, the Roe C*-algebra $\textrm{C}^*_\textrm{Roe}(\Lambda )$ has simple K-theory groups. It follows from coarse Mayer–Vietoris sequence (cf. [13]) that the K-theory of $\textrm{C}^*_\textrm{Roe}(\mathbb {R}^d)$ coincides with the K-theory of a point by a degree shift of $-d$. This holds for any Delone set $\Lambda \subseteq \mathbb {R}^d$ as well by Proposition 3.22. Therefore, K-theory of the real or complex Roe C*-algebras of a Delone set $\Lambda \subseteq \mathbb {R}^d$ is given by

3.24 $$\begin{aligned} \begin{aligned} \operatorname {K}_i(\textrm{C}^*_\textrm{Roe}(\Lambda )_\mathbb {C})&\simeq {\left\{ \begin{array}{ll} {{\mathbb {Z}}/2} &  \text {if } i-d\equiv 0\,\,\,\mod 2; \\ 0 &  \text {if } i-d\equiv 1\,\,\,\mod 2. \end{array}\right. } \\ \operatorname {K}_i(\textrm{C}^*_\textrm{Roe}(\Lambda )_\mathbb {R})&\simeq {\left\{ \begin{array}{ll} \mathbb {Z}&  \text {if } i-d\equiv 0,4\,\,\,\mod 8; \\ \mathbb {Z}/2 &  \text {if } i-d\equiv 1,2\,\,\,\mod 8; \\ 0 &  \text {if } i-d\equiv 3,5,6,7\,\mod 8. \end{array}\right. } \end{aligned} \end{aligned}$$

We note that the isomorphisms of abelian groups in (3.24) are actually given by $\operatorname {K}_*(\mathbb {C})$-module or $\operatorname {KO}_*(\mathbb {R})$-module isomorphisms

$$\begin{aligned} \operatorname {K}_*(\textrm{C}^*_\textrm{Roe}(\Lambda )_\mathbb {C})\simeq \operatorname {K}_{*-d}(\mathbb {C}),\quad \operatorname {KO}_*(\textrm{C}^*_\textrm{Roe}(\Lambda )_\mathbb {R})\simeq \operatorname {KO}_{*-d}(\mathbb {R}), \end{aligned}$$

cf. ( [12], Section 3 and Corollary 5).

While using the Roe C*-algebra as the observable C*-algebra, there is no need to pass to its matrix algebras, as opposed to the case of groupoids. It was proven in ( [12], Corollary 1) that an operator $T$ on $\mathcal {H}_\Lambda ^{\oplus N}$ is locally compact and controlled if and only if its matrix elements in $\mathbb {B}(\mathcal {H}_\Lambda )$ are locally compact and controlled, when $T$ is viewed as an $N\times N$-matrix with entries in $\mathbb {B}(\mathcal {H}_\Lambda )$. Therefore

$$\begin{aligned} \mathbb {M}_N\textrm{C}^*_\textrm{Roe}(\Lambda ,\mathcal {H}_\Lambda )\simeq \textrm{C}^*_\textrm{Roe}(\Lambda ,\mathcal {H}_\Lambda ^{\oplus N}) \end{aligned}$$

for all $N$, where $\mathcal {H}_\Lambda ^{\oplus N}$ carries the direct sum of the standard ample representation $\textrm{C}_0(\Lambda )\rightarrow \mathbb {B}(\mathcal {H}_\Lambda )$. We may further identify $\mathcal {H}_\Lambda ^{\oplus }\simeq \ell ^2(\Lambda ,\mathcal {K})\otimes \mathbb {R}^N\simeq \ell ^2(\Lambda ,\mathcal {K}\otimes \mathbb {R}^N)\simeq \ell ^2(\Lambda ,\mathcal {K})$ using any unitary isomorphism $\mathcal {K}\simeq \mathcal {K}\otimes \mathbb {R}^N$. Such an isomorphism preserves controlled or locally compact operators using the characterisation in Example 3.18.

#### Position spectral triple over the Roe C*-algebra

Fix a closed subset $X\subseteq \mathbb {R}^d$ equipped with the subspace metric, e.g. a Delone set in $\mathbb {R}^d$. A relation between the Roe C*-algebra on $X$ and the “position” operators has been considered in ( [12], Section 2.2) as follows. For $t=(t_1,\dots ,t_d)\in \mathbb {R}^d$, let $\textrm{e}_t$ be the bounded continuous function on $X\subseteq \mathbb {R}^d$ given by

$$\begin{aligned} \textrm{e}_t(x)\mathrel {:=}\textrm{e}^{\textrm{i}t\cdot x}=\textrm{e}^{\textrm{i}\sum _{j=1}^dt_jx_j},\qquad x=(x_1,\dots ,x_d)\in X\subseteq \mathbb {R}^d. \end{aligned}$$

Then the map $\mathbb {R}^d\rightarrow \textrm{C}_\textrm{b}(X)$ given by $t\mapsto \textrm{e}_t$ is continuous in the strict topology of $\textrm{C}_\textrm{b}(X)$.

Let $\mathcal {H}_X$ be an ample $X$-module. The representation $\varrho :\textrm{C}_0(X)\rightarrow \mathbb {B}(\mathcal {H}_X)$ extends to a strictly continuous, *-representation $\overline{\varrho }:\textrm{C}_\textrm{b}(X)\rightarrow \mathbb {B}(\mathcal {H}_X)$. Thus the map

$$\begin{aligned} \sigma :\mathbb {R}^d\rightarrow \textrm{C}_\textrm{b}(X)\rightarrow \mathbb {B}(\mathcal {H}_X),\qquad t\mapsto \textrm{e}_t\mapsto \varrho (\textrm{e}_t) \end{aligned}$$

is continuous for the norm topology on $\mathbb {B}(\mathcal {H}_X)$. If $X$ does not contain discrete components, and $\mathcal {H}_X$ is the standard $X$-module as in Example 3.15, then the restriction of $\sigma$ to the $j$-th coordinate component of $\mathbb {R}^d$ gives the flow  generated by .

By conjugation, $\sigma :\mathbb {R}^d\rightarrow \mathbb {B}(\mathcal {H}_X)$ induces a group homomorphism $\operatorname {Ad}\sigma :\mathbb {R}^d\rightarrow \operatorname {Aut}(\mathbb {B}(\mathcal {H}_X))$. We say that an operator $T\in \mathbb {B}(\mathcal {H}_X)$ is continuous with respect to $\operatorname {Ad}\sigma$ if and only if the map $t\mapsto \operatorname {Ad}\sigma _t(T)$ is continuous for the norm topology on $\mathbb {B}(\mathcal {H})$. The property of being a norm limit of controlled operators can be described as a continuity property for the action $\operatorname {Ad}\sigma$:

##### Lemma 3.25

([12], Theorem 4) An operator $T\in \mathbb {B}(\mathcal {H}_X)$ is a norm limit of controlled operators if and only if it is continuous with respect to $\operatorname {Ad}\sigma$. Thus $T\in \textrm{C}^*_\textrm{Roe}(X,\mathcal {H}_X)$ if and only if it is locally compact and continuous with respect to $\operatorname {Ad}\sigma$.

We take a slight different viewpoint, interpreting the previous lemma as follows: the Roe C*-algebra is the largest C*-algebra, over which one can have a position spectral triple:

##### Theorem 3.26

Let $\Lambda \subseteq \mathbb {R}^d$ be a discrete subset. Ifis a position spectral triple (cf. Definition 2.11), where $\mathcal {A}$ is a dense *-subalgebra of a C*-algebra $A$ represented on $\ell ^2(\Lambda )\otimes \mathcal {K}$ by $\varphi :A\rightarrow \mathbb {B}(\ell ^2(\Lambda )\otimes \mathcal {K})$. Then $\varphi (\mathcal {A})$ is contained in the Roe C*-algebra $\textrm{C}^*_\textrm{Roe}(\Lambda ,\mathcal {H}_\Lambda )$ defined by the standard $\Lambda$-module $\mathcal {H}_\Lambda \mathrel {:=}\ell ^2(\Lambda )\otimes \mathcal {K}$ as in Example 3.15.

##### Proof

Identifying $A$ and its dense subalgebra $\mathcal {A}$ with their images under $\varphi$, we may assume that $\varphi :A\rightarrow \mathbb {B}(\ell ^2(\Lambda )\otimes \mathcal {K})$ is injective. Then we must show that every $a\in \mathcal {A}$ is locally compact and can be approximated by controlled operators. Being locally compact amounts to saying that  for all $x,y\in \Lambda$, which always holds by Lemma 2.13 (providing that $\xi$ *is* a position spectral triple.)

Now we show that every $a\in \mathcal {A}$ is a norm limit of controlled operators. For convenience, below we write  for the operator  on $\ell ^2(\Lambda )\otimes \mathcal {K}$.

Since  is bounded for all $a\in \mathcal {A}$ and $i,j\in \{1,\dots ,d\}$, we have  is a bounded operator for all $j\in \{1,\dots ,d\}$, i.e. there exists $C>0$ such that .

By Lemma 3.25, it suffices to show that for all $a\in \mathcal {A}$ and $j=1,\dots ,d$, the mapis continuous in the norm topology on $\mathbb {B}(\ell ^2(\Lambda ,\mathcal {K}))$. It follows fromthatTherefore,So the map $t\mapsto \operatorname {Ad}{\sigma _t}(a)$ is continuous for all $a\in \mathcal {A}$, and thus $a\in \mathcal {A}$ is a norm limit of controlled operators. $\square$

Thus if the *-algebra $\mathcal {A}$ in the position spectral triple is dense in $\textrm{C}^*_\textrm{Roe}(\Lambda )$, then it defines a spectral triple over $\textrm{C}^*_\textrm{Roe}(\Lambda )$. This amounts to choosing a suitable dense *-subalgebra inside $\textrm{C}^*_\textrm{Roe}(\Lambda )$. The spectral triple below has been implicitly used by Ewert and Meyer in the proof of ( [12], Theorem 7).

##### Definition and Lemma 3.27

Let $\Lambda \subseteq \mathbb {R}^d$ be a discrete countable set. Let $\mathcal {C}_\textrm{Roe}(\Lambda )$ be the collection of operators $T\in \mathbb {B}(\ell ^2(\Lambda ,\mathcal {K}))$ satisfying:

- $T$ is controlled and locally compact;
- $$ \sup _{y\in \Lambda }\sum _{x\in \Lambda }\left\Vert T_{x,y}\right\Vert <+\infty $$ and $$ \sup _{x\in \Lambda }\sum _{y\in \Lambda }\left\Vert T_{x,y}\right\Vert <+\infty $$.

Then3.28is a position spectral triple over $\textrm{C}^*_\textrm{Roe}(\Lambda )$.

##### Proof

Let $T\in \mathcal {C}_\textrm{Roe}(\Lambda )$, $x,y\in \Lambda$. ThenSince $T$ is controlled, there exists $R>0$ such that  whenever $\textrm{d}(x,y)>R$. ThusSo $T$ preserves the domain of  and boundedly commutes with it for every $j=1,\dots ,d$.

Therefore, for any $S\in \textrm{C}\ell _{0,d}$, $T\otimes S$ preserves the domain of , and boundedly commutes with . The local compactness of $T$ implies that the operatoris compact. Therefore, $\xi ^\textrm{Roe}_\Lambda$ is a position spectral triple. $\square$

#### Position spectral triple generates $\operatorname {KO}_*(\mathbb {R})$

Towards the end of this section, we shall prove that the position spectral triple $\xi _\Lambda ^\textrm{Roe}$ induces isomorphisms

$$\begin{aligned} \operatorname {KK}(\textrm{C}\ell _{n,0},\textrm{C}^*_\textrm{Roe}(\Lambda ))\xrightarrow {\sim }\operatorname {KK}(\textrm{C}\ell _{n,d},\mathbb {R}) \end{aligned}$$

for all $n$. The proof is based on these observations.

1. If $\Lambda =\mathbb {Z}^d$, then the KK-class of the position spectral triple
  $$\begin{aligned} [\xi ^\textrm{Roe}_{\mathbb {Z}^d}]\in \operatorname {KK}(\textrm{C}^*_\textrm{Roe}(\mathbb {Z}^d)_\mathbb {R}\otimes \textrm{C}\ell _{0,d},\mathbb {R}) \end{aligned}$$
  pulls back to the fundamental class of $\operatorname {KK}(\textrm{C}^*(\mathbb {Z}^d)_\mathbb {R}\otimes \textrm{C}\ell _{0,d},\mathbb {R})$ as in Theorem 2.15. Therefore, for each $n$, it induces an isomorphism
  $$\begin{aligned} \operatorname {KK}(\textrm{C}\ell _{n,0},\textrm{C}^*_\textrm{Roe}(\mathbb {Z}^d))\xrightarrow {\sim }\operatorname {KK}(\textrm{C}\ell _{n,d},\mathbb {R}). \end{aligned}$$
2. Any other Delone set $\Lambda \subseteq \mathbb {R}^d$ is coarsely equivalent to $\mathbb {Z}^d$. Therefore, $\xi ^\textrm{Roe}_{\Lambda }$ induces the same map as $\xi ^\textrm{Roe}_{\mathbb {Z}^d}$ up to the isomorphism given by
  $$\begin{aligned} \operatorname {KK}(\textrm{C}\ell _{n,0},\textrm{C}^*_\textrm{Roe}(\mathbb {Z}^d))\simeq \operatorname {KK}(\textrm{C}\ell _{n,0},\textrm{C}^*_\textrm{Roe}(\Lambda )). \end{aligned}$$

##### Lemma 3.29

The position spectral triple for $\Lambda =\mathbb {Z}^d$3.30induces, for each $n$, an isomorphism

$$\begin{aligned} \operatorname {KK}(\textrm{C}\ell _{n,0},\textrm{C}^*_\textrm{Roe}(\mathbb {Z}^d))\xrightarrow {\sim }\operatorname {KK}(\textrm{C}\ell _{n,d},\mathbb {R}). \end{aligned}$$

##### Proof

The proof comes from ( [12], Theorem 7, Corollary 5), which we sketch here.

First let $n=d$ and $\iota :\textrm{C}^*(\mathbb {Z}^d)\hookrightarrow \textrm{C}^*_\textrm{Roe}(\mathbb {Z}^d)$ be the inclusion map induced by the obvious isometry $\ell ^2(\mathbb {Z}^d)\hookrightarrow \ell ^2(\mathbb {Z}^d)\otimes \mathcal {K}$. Then

$$\begin{aligned} \xi _{\mathbb {Z}^d,1}=\iota ^*\xi _{\mathbb {Z}^d}^\textrm{Roe}, \end{aligned}$$

that is, $\xi _{\mathbb {Z}^d,1}$ is the pullback of the position spectral triple over $\textrm{C}^*_\textrm{Roe}(\mathbb {Z}^d)_\mathbb {R}$. Thus the following diagram commutes by functorality of the Kasparov product (cf. Lemma 2.10):
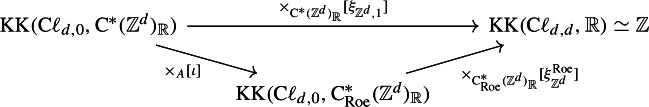


and the horizontal arrow is a surjective group homomorphism by Theorem 2.15. Thus the bottom right arrow is a surjective group homomorphism $\mathbb {Z}\rightarrow \mathbb {Z}$, hence an isomorphism.

The isomorphisms of abelian groups $\operatorname {KO}_n(\textrm{C}^*_\textrm{Roe}(\mathbb {Z}^d)_\mathbb {R})\simeq \operatorname {KO}_{n-d}(\mathbb {R})$ give a give an isomorphism of $\operatorname {KO}_*(\mathbb {R})$-modules $\operatorname {KO}_*(\textrm{C}^*_\textrm{Roe}(\mathbb {Z}^d)_\mathbb {R})\simeq \operatorname {KO}_{*-d}(\mathbb {R})$ (cf. the discussion after ( [12], Proposition 8)). Functorality of the Kasparov product implies that $\xi _{\mathbb {Z}^d}^\textrm{Roe}$ induces a $\operatorname {KO}_*(\mathbb {R})$-module homomorphism $\operatorname {KO}_*(\textrm{C}^*_\textrm{Roe}(\mathbb {Z}^d)_\mathbb {R})\rightarrow \operatorname {KO}_{*-d}(\mathbb {R})$, both sides being isomorphic to free $\operatorname {KO}_*(\mathbb {R})$-modules with a generator in degree $-d$. The commutative diagram above shows that $[\xi _{\mathbb {Z}^d}^\textrm{Roe}]$ maps such a generator to a generator, hence a $\operatorname {KO}_*(\mathbb {R})$-module isomorphism. In particular, it follows that the induced maps

$$\begin{aligned} \xi _{\mathbb {Z}^d}^\textrm{Roe}:\underbrace{\operatorname {KK}(\textrm{C}\ell _{n,0},\textrm{C}^*_\textrm{Roe}(\mathbb {Z}^d)_\mathbb {R})}_{\simeq \operatorname {KO}_n(\textrm{C}^*_\textrm{Roe}(\mathbb {Z}^d)_\mathbb {R})}\rightarrow \underbrace{\operatorname {KK}(\textrm{C}\ell _{n,d},\mathbb {R})}_{\simeq \operatorname {KO}_{n-d}(\mathbb {R})} \end{aligned}$$

are isomorphisms of abelian groups for all $n$. $\square$

##### Lemma 3.31

Let $L_1,L_2\subseteq \mathbb {R}^d$ be Delone sets such that $L_1\cap L_2=\emptyset$. Let $L\mathrel {:=}L_1\sqcup L_2$. For $i\in \{1,2\}$, we write:

- $\mathcal {H}_i\mathrel {:=}\ell ^2(L_i,\mathcal {K})$ for the standard ample $L_i$-module, and $\mathcal {H}\mathrel {:=}\ell ^2(L_1\sqcup L_2,\mathcal {K})$ for the standard ample $L$-module;
- $\xi ^\textrm{Roe}_{L_i}$ and $\xi ^\textrm{Roe}_L$ for the position spectral triples over $\textrm{C}^*_\textrm{Roe}(L_i)$ and $\textrm{C}^*_\textrm{Roe}(L)$ as in (3.28);
- $\iota _i:L_i\hookrightarrow L$ for the isometric embeddings;
- $V_i:\mathcal {H}_i\hookrightarrow \mathcal {H}_0$ for the isometries induced by $\iota _i$, that is, $V_i|x\rangle \mathrel {:=}|\iota _i(x)\rangle$ for all $x\in L_i$.

Then the following hold:

1. $V_i's$ are covering isometries for $\iota _i$. Hence $\operatorname {Ad}_{V_i}:T\mapsto V_iTV_i^*$ maps $\textrm{C}^*_\textrm{Roe}(L_i)$ into $\textrm{C}^*_\textrm{Roe}(L)$, and induces an isomorphism
  $$\begin{aligned} \iota _{i*}:\operatorname {KK}(\textrm{C}\ell _{n,0},\textrm{C}^*_\textrm{Roe}(L_i))\rightarrow \operatorname {KK}(\textrm{C}\ell _{n,0},\textrm{C}^*_\textrm{Roe}(L)). \end{aligned}$$
2. The following diagram commutes (all arrows are given by taking the Kasparov product): 3.32

##### Proof

(1) follows from Proposition 3.22 as both $L_1$ and $L_2$ are coarsely dense in $L$. Since $L_1\cap L_2=\emptyset$ and $L=L_1\sqcup L_2$, it follows that

$$\begin{aligned} \mathcal {H}_1\oplus \mathcal {H}_2\xrightarrow {\sim }\mathcal {H},\quad (\phi _1,\phi _2)\mapsto V_1\phi _1+V_2\phi _2. \end{aligned}$$

is a unitary isomorphism of Hilbert spaces.

Let  be the $j$-th position operator on $\ell ^2(L_i)$, that is,Also write  for the $j$-th position operator on $\ell ^2(L)$. Then  restricts to the $j$-th position operator on $\ell ^2(L_i)$. That is, we haveand henceTherefore, for $i\in \{1,2\}$, the *-homomorphism $\operatorname {Ad}_{V_{j}}$ pulls back the class $[\xi _L^\textrm{Roe}]\in \operatorname {KK}(\textrm{C}^*_\textrm{Roe}(L)\otimes \textrm{C}\ell _{0,d},\mathbb {R})$ to the class $[\xi _{L_i}^\textrm{Roe}]\in \operatorname {KK}(\textrm{C}^*_\textrm{Roe}(L_i)\otimes \textrm{C}\ell _{0,d},\mathbb {R})$. So both the left and the right triangle commutes by Lemma 2.10. $\square$

##### Theorem 3.33

For any Delone set $\Lambda$, the position spectral triple $\xi ^\textrm{Roe}_\Lambda$ induces, for each $n$, an isomorphism

$$\begin{aligned} \operatorname {KK}(\textrm{C}\ell _{n,0},\textrm{C}^*_\textrm{Roe}(\Lambda ))\xrightarrow {\sim }\operatorname {KK}(\textrm{C}\ell _{n,d},\mathbb {R}). \end{aligned}$$

##### Proof

The first step is to replace $\Lambda$ by its translated copy that is disjoint from $\mathbb {Z}^d$. To be precise, we note that the set

$$\begin{aligned} B\mathrel {:=}\lbrace x-y\mid x\in \Lambda ,y\in \mathbb {Z}^d\rbrace \end{aligned}$$

is countable, so the set $\mathbb {R}^d\setminus B$ is non-empty. Choose any $v\in \mathbb {R}^d\setminus B$, then the Delone set $\Lambda '\mathrel {:=}\Lambda -v$ is disjoint from $\mathbb {Z}^d$. The translation map

$$\begin{aligned} f:\Lambda \rightarrow \Lambda ',\quad x\mapsto x-v \end{aligned}$$

induces a unitary isomorphism of Hilbert spaces $V:\ell ^2(\Lambda )\xrightarrow {\sim }\ell ^2(\Lambda ')$ that covers $f$. Hence $V$ gives a *-isomorphism $\operatorname {Ad}_V:\textrm{C}^*_\textrm{Roe}(\Lambda )\xrightarrow {\sim }\textrm{C}^*_\textrm{Roe}(\Lambda ')$, which pulls back the position spectral triple $\xi _{\Lambda '}^\textrm{Roe}$ to the position spectral triple $\xi _{\Lambda }^\textrm{Roe}$.

As a consequence, we may assume without loss of generality that $\mathbb {Z}^d\cap \Lambda =\emptyset$. Set $L_1\mathrel {:=}\mathbb {Z}^d$ and $L_2\mathrel {:=}\Lambda$. Then the position spectral triple $\xi ^\textrm{Roe}_{L_1}=\xi ^\textrm{Roe}_{\mathbb {Z}^d}$ induces an isomorphism

$$\begin{aligned} \operatorname {KK}(\textrm{C}\ell _{n,0},\textrm{C}^*_\textrm{Roe}(\mathbb {Z}^d))\xrightarrow {\sim }\operatorname {KK}(\textrm{C}\ell _{n,d},\mathbb {R}) \end{aligned}$$

by Lemma 3.29. The isometry $V_{1}$ induces an isomorphism between the corresponding Kasparov groups by Lemma 3.31. Thus the commutativity of the left triangle in 3.32 implies that the map

$$\begin{aligned} \operatorname {KK}(\textrm{C}\ell _{n,0},\textrm{C}^*_\textrm{Roe}(\mathbb {Z}^d\sqcup \Lambda ))\rightarrow \operatorname {KK}(\textrm{C}\ell _{n,d},\mathbb {R}) \end{aligned}$$

given by the position spectral triple $\xi ^\textrm{Roe}_{\mathbb {Z}^d\sqcup \Lambda }$, must be an isomorphism. Thus the position spectral triple $\xi _{L_2}^\textrm{Roe}=\xi _\Lambda ^\textrm{Roe}$ must be an isomorphism as well, as it is a composition of isomorphisms

$$\begin{aligned} \operatorname {KK}(\textrm{C}\ell _{n,0},\textrm{C}^*_\textrm{Roe}(\Lambda )) \underset{\sim }{\xrightarrow {\iota _{1*}}}\operatorname {KK}(\textrm{C}\ell _{n,0},\textrm{C}^*_\textrm{Roe}(\mathbb {Z}^d\sqcup \Lambda )) \underset{\sim }{\xrightarrow {\xi _{L}^\textrm{Roe}}}\operatorname {KK}(\textrm{C}\ell _{n,d},\mathbb {R}). \end{aligned}$$

$$\square $$

#### Remarks on uniform Roe C*-algebras

Let $\Lambda$ be a discrete metric space. An operator $T\in \mathbb {B}(\ell ^2(\Lambda ))$ is called *controlled*, if there exists $R>0$ such that  whenever $x,y\in \Lambda$ satisfy $\textrm{d}(x,y)>R$. The *uniform Roe* C*-*algebra* of $\Lambda$, denoted by $\textrm{C}^*_{\textrm{u,Roe}}(\Lambda )$, is the C*-algebra generated by all controlled operators on $\ell ^2(\Lambda )$.

Starting from Kubota [19], uniform Roe C*-algebras have also been employed as the observable C*-algebras of aperiodic topological insulators. As opposed to Roe C*-algebras, uniform Roe C*-algebras have much more involved K-theory. For example, it is known that $\operatorname {K}_0(\textrm{C}^*_{\textrm{u,Roe}}(\mathbb {Z}))$ is uncountable for $d\ge 1$ (cf. [26], Example II.3.4).

We briefly remark here that the uniform Roe C*-algebra is also quite “universal” as it contains all “tight-binding” observable C*-algebras, in the following sense:

##### Proposition 3.34

Assume that there is a real spectral triple of the form3.35where:

- $A$ is a real C*-algebra, which carries a *-representation $\varphi :A\rightarrow \mathbb {B}(\ell ^2(\Lambda ))$; $\mathcal {A}\subseteq A$ is a dense *-subalgebra;
is the $j$-th position operator on $\ell ^2(\Lambda )$, given by
- $\gamma _1,\dots ,\gamma _d$ are the generators of $\textrm{C}\ell _{d,0}$, represented on $\bigwedge \nolimits ^*\mathbb {R}^{d}$ via the standard representation.

Then $\varphi (A)$ is contained in $\textrm{C}^*_{\textrm{u,Roe}}(\Lambda )$.

We note that the auxiliary Hilbert space $\mathcal {K}$ is now absent, as opposed to the definition of a position spectral triple (cf. Definition 2.11). Thus $A$ is merely represented on the “tight-binding” Hilbert space $\ell ^2(\Lambda )$ instead of $\ell ^2(\Lambda )\otimes \mathcal {K}$. Replacing $A$ by $A\otimes \mathbb {M}_N(\mathbb {R})$ replaces $\ell ^2(\Lambda )$ by $\ell ^2(\Lambda ,\mathbb {R}^N)=\ell ^2(\Lambda )\otimes \mathbb {R}^N$.

##### Proof

It follows from Lemma 3.25 and the proof of Theorem 3.26 that given the spectral triple $\zeta _\Lambda$, then every $a\in \mathcal {A}$ is represented as a controlled operator on $\ell ^2(\Lambda )$. Thus $\varphi (\mathcal {A})\subseteq \textrm{C}^*_{\textrm{u,Roe}}(\Lambda )$ and hence $\varphi (A)\subseteq \textrm{C}^*_{\textrm{u,Roe}}(\Lambda )$. $\square$

Thus tight-binding models of topological insulators, e.g. the groupoid model $\textrm{C}^*(\mathcal {G}_\Lambda )$, is already contained in the uniform Roe C*-algebra $\textrm{C}^*_{\textrm{u,Roe}}(\Lambda )$, and we have inclusions of C*-algebras and isometric embeddings of Hilbert spaces which they act on:
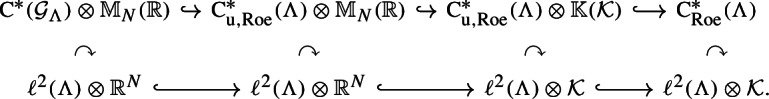


As argued in ( [12], Section 1), the occurrence of uniform Roe C*-algebras might be considered as an artefact of the tight-binding approximation.

## Robustness of topological phases

Now we use the results from previous sections to compare the groupoid model $\textrm{C}^*(\mathcal {G}_\Lambda )$ with the coarse-geometric model $\textrm{C}^*_\textrm{Roe}(\Lambda )$ of topological phases on an aperiodic lattice $\Lambda$. Topological phases described by the K-theory of $\textrm{C}^*_\textrm{Roe}(\Lambda )$ are called *strong* in [12]. We follow this terminology. Such phases are stable under perturbations which perserve the conjugate-linear and/or chiral symmtries of the system, are locally compact and controlled, and do not close the gap of the Hamiltonian. This is because such perturbations lift to homotopies in the Roe C*-algebra, and hence preserve K-theory.

We will explain which phases, described by the K-theory of $\textrm{C}^*(\mathcal {G}_\Lambda )$, have the same robustness. This is done by mapping $\textrm{C}^*(\mathcal {G}_\Lambda )$ into a Roe C*-algebra $\textrm{C}^*_\textrm{Roe}(\omega )$ using the localised regular representations $\pi _\omega$ depending on a choice of $\omega \in \Omega$, whereas all of these $\omega$ yield isomorphic Roe C*-algebras as they are Delone sets in $\mathbb {R}^d$. We also explain that “stacked” topological phases, coming from lower-dimensional Delone sets, are always weak. Both results can be viewed as generalisations of ( [12], Section 4), in which the Delone set $\Lambda$ is the periodic square lattice $\mathbb {Z}^d$.

### Position spectral triples detect strong topological phases

The results of Theorem 3.12 and Theorem 3.33 allows us to compare the groupoid model and the coarse-geometric model on the level of both C*-algebras and K-theory (index pairing). The main theorem is the following:

#### Theorem 4.1

(Position spectral triples detect strong topological phases) For every $\omega \in \Omega _0$, The following diagram commutes:4.2where:

- $\pi _\omega ^N\mathrel {:=}\pi _\omega \otimes e_N:\textrm{C}^*(\mathcal {G}_\Lambda )\otimes \mathbb {M}_N(\mathbb {R})\rightarrow \mathbb {B}(\ell ^2(\omega )\otimes \mathcal {K})$ is the entrywise extension of the localised regular representation at $\omega \in \Omega _0$ as in (3.10);
- $_{d}{\lambda }_{\Omega _0}$ is the bulk cycle as in (3.11);
- $\xi ^\textrm{Gpd}_{\omega ,N}$ is the spectral triple over $\textrm{C}^*(\mathcal {G}_\Lambda )\otimes \mathbb {M}_N(\mathbb {R})$ defined in (3.13); $\xi ^\textrm{Roe}_\omega$ is the spectral triple over $\textrm{C}^*_\textrm{Roe}(\omega )$ defined in (3.28);
- all arrows are given by taking Kasparov products.

#### Proof

We claim that $\pi ^N_\omega :\textrm{C}^*(\mathcal {G}_\Lambda )\otimes \mathbb {M}_N(\mathbb {R}) \rightarrow \mathbb {B}(\ell ^2(\omega )\otimes \mathcal {K})$ maps into $\textrm{C}^*_\textrm{Roe}(\omega )$ defined by the standard ample $\omega$-module $\ell ^2(\omega ,\mathcal {K})$. Whenever this holds, then it follows that the KK-class represented by the spectral triple (3.13)equals the pullback along $\pi ^N_\omega$ of the KK-class represented by (3.28):We have shown in Theorem 3.12 that $\xi ^\textrm{Gpd}_{\omega ,N}$ is a position spectral triple. This implies that $\pi _\omega (\textrm{C}_\textrm{c}(\mathcal {G}_\Lambda )\otimes \mathbb {M}_N(\mathbb {R})) \subseteq \textrm{C}^*_\textrm{Roe}(\Lambda )$ by Theorem 3.26. Since $\textrm{C}_\textrm{c}(\mathcal {G}_\Lambda )\otimes \mathbb {M}_N(\mathbb {R})$ is dense in $\textrm{C}^*(\mathcal {G}_\Lambda )\otimes \mathbb {M}_N(\mathbb {R})$, the image of $\pi _\omega ^N$ is contained in $\textrm{C}^*_\textrm{Roe}(\omega )$. Then it follows from the functoriality of the Kasparov product in Lemma 2.10 that the bottom left triangle commutes.

The commutativity of the top right triangle follows from the construction of $\xi ^\textrm{Gpd}_\omega$ in Theorem 3.12. Therefore, we conclude that the entire diagram commutes. $\square$

#### Example 4.3

Consider the periodic square lattice $\Lambda =\mathbb {Z}^d$. Then $\Omega _\Lambda \simeq \mathbb {T}^d$ and $\Omega _0=\{\omega \}$ is a singleton, in which $\omega$ can be chosen to be any point in $\Omega _\Lambda$ (cf. ( [9], Example 2.7)). There is a unique evaluation point $\operatorname {ev}_\omega :\{\omega \}\rightarrow \mathbb {C}$, which gives an isomorphism $\operatorname {KO}^0(\operatorname {pt})=\operatorname {KO}_0(\mathbb {R})\xrightarrow {\sim }\mathbb {Z}$. The spectral triple $\xi ^\textrm{Gpd}_{\omega ,N}$ is nothing but the position spectral triple $\xi _{\mathbb {Z}^d,N}$ over the real group C*-algebra $\textrm{C}^*(\mathbb {Z}^d)_\mathbb {R}$ in (2.14). And the diagram 4.2 recovers the commutative diagram in ( [12], Theorem 7).

### Stacked topological phases are weak

It was explained in [12] why certain topological phases described by $\operatorname {K}_*(\textrm{C}^*(\mathbb {Z}^d))$ are “weak”. Let $\varphi :\mathbb {Z}^{d}\rightarrow \mathbb {Z}^{d+1}$ is an injective group homomorphism. It induces a map $\varphi _*:\textrm{C}^*(\mathbb {Z}^{d})\rightarrow \textrm{C}^*(\mathbb {Z}^{d+1})$ and hence maps

$$\begin{aligned} \varphi _*:\operatorname {K}_*(\textrm{C}^*(\mathbb {Z}^{d}))\rightarrow \operatorname {K}_*(\textrm{C}^*(\mathbb {Z}^{d+1})) \end{aligned}$$

in K-theory. Topological phases that belong to the image of $\varphi _*$ can be thought of as “stacked” from the lower-dimensional lattice $\mathbb {Z}^d$. It was shown in ( [12], Proposition 10) that such phases are killed by the map $\operatorname {K}_*(\textrm{C}^*(\mathbb {Z}^{d+1}))\rightarrow \operatorname {K}_*(\textrm{C}^*_\textrm{Roe}(\mathbb {Z}^{d+1}))$ induced by the inclusion $\textrm{C}^*(\mathbb {Z}^{d+1})\hookrightarrow \textrm{C}^*_\textrm{Roe}(\mathbb {Z}^{d+1})$. The proof is based on the fact that $\varphi$ also induces a map between the Roe C*-algebras of $\mathbb {Z}^d$ and $\mathbb {Z}^{d+1}$, which factors through a flasque space if $\varphi$ is injective. Then this map induces zero maps in K-theory.

**Fig. 2:**
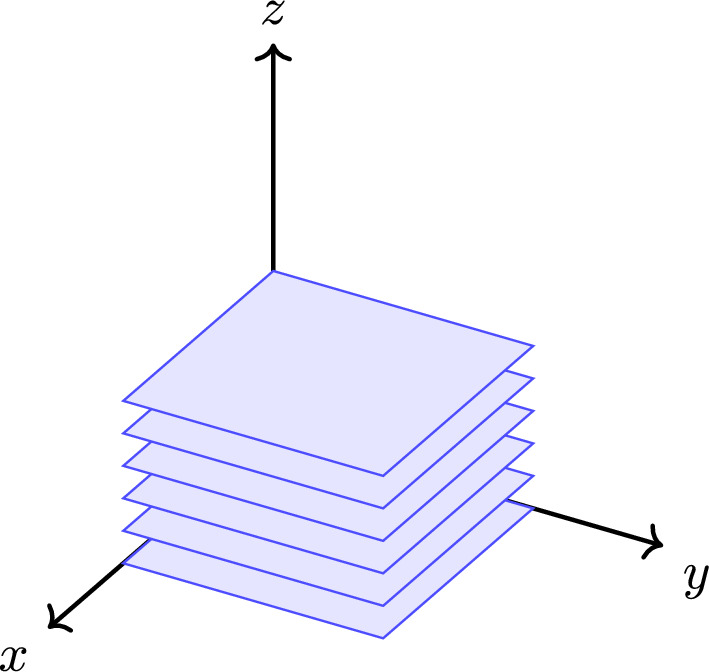
“Stacked” topological phases along the $z$-direction

We shall explain in this section how this observation can be generalised to aperiodic lattices and higher-dimensional cases. That is, we consider Delone sets of the form $\Lambda \times L$, where $L$ is another Delone set. If $L$ has dimension $1$, then we may think of $\Lambda \times L$ as “stacking” $\Lambda$ along the direction of $L$ (cf. Figure 2). This also gives a *-homomorphism groupoid *-homomorphism $\varphi ^\textrm{Gpd}:\textrm{C}^*(\mathcal {G}_\Lambda ) \rightarrow \textrm{C}^*(\mathcal {G}_{\Lambda \times L})$, which induces maps

$$\begin{aligned} \varphi ^\textrm{Gpd}_*:\operatorname {KK}(\textrm{C}\ell _{n,0},\textrm{C}^*(\mathcal {G}_\Lambda ))\rightarrow \operatorname {KK}(\textrm{C}\ell _{n,0},\textrm{C}^*(\mathcal {G}_{\Lambda \times L})) \end{aligned}$$

for all $n$. Such maps can be interpreted as “stacking” topological phases living on $\Lambda$ along the direction $L$. We will show that such topological phases are always weak, in the sense that they vanish in the K-theory of Roe C*-algebras.

We fix the following notation. Let $\Lambda \subseteq \mathbb {R}^p$ be an $(r_\Lambda ,R_\Lambda )$-Delone set and $L\subseteq \mathbb {R}^q$ be an $(r_L,R_L)$-Delone set. We write $\Omega _{\Lambda \times L}$, $\Omega _{\Lambda }$ and $\Omega _L$ for the closure of the orbit of $\Lambda \times L$, $\Lambda$ and $L$. To distinguish, we write

$$\begin{aligned} \Omega ^{\Lambda \times L}_0\mathrel {:=}\lbrace \mu \in \Omega _{\Lambda \times L}\mid 0\in \mu \rbrace ,\quad \Omega ^\Lambda _0\mathrel {:=}\lbrace \omega \in \Omega _{\Lambda }\mid 0\in \omega \rbrace ,\quad \Omega ^L_0\mathrel {:=}\left\rbrace \ell \in \Omega _L\;\big |\;0\in \ell \right\lbrace \end{aligned}$$

for corresponding abstract transversals.

#### Proposition 4.4

The set

$$\begin{aligned} \Lambda \times L\mathrel {:=}\left\rbrace (x,a)\;\big |\;x\in \Lambda ,a\in L\right\lbrace \subseteq \mathbb {R}^{p+q} \end{aligned}$$

is also a Delone set.

#### Proof

Choose $0<r<R$ satisfying $r<\min \{r_L,r_{\Lambda }\}$ and $R>\sqrt{R_\Lambda ^2+R_L^2}$. Then for all $(x,a)\in \mathbb {R}^m\times \mathbb {R}^n$, we have

$$\begin{aligned} \operatorname {B}((x,a),r)\subseteq \operatorname {B}(x,r_\Lambda )\times \operatorname {B}(a,r_L),\quad \operatorname {B}((x,a),R)\supseteq \operatorname {B}(x,R_\Lambda )\times \operatorname {B}(a,R_L). \end{aligned}$$

Hence

$$\begin{aligned} \#(\operatorname {B}((x,a),r)\cap (\Lambda \times L))&\le \#((\operatorname {B}(x,r_\Lambda )\times \operatorname {B}(a,r_L))\cap (\Lambda \times L)) \\&=\#((\operatorname {B}(x,r_\Lambda )\cap \Lambda )\times (\operatorname {B}(a,r_L)\cap L)) \\&=\#(\operatorname {B}(x,r_\Lambda )\cap \Lambda )\cdot \#(\operatorname {B}(a,r_L)\cap L) \\&\le 1,\\ \#(\operatorname {B}((x,a),R)\cap (\Lambda \times L))&\ge \#((\operatorname {B}(x,R_\Lambda )\times \operatorname {B}(a,R_L))\cap (\Lambda \times L)) \\&=\#((\operatorname {B}(x,R_\Lambda )\cap \Lambda )\times (\operatorname {B}(a,R_L)\cap L)) \\&=\#(\operatorname {B}(x,R_\Lambda )\cap \Lambda )\cdot \#(\operatorname {B}(a,R_L)\cap L) \\&\ge 1. \end{aligned}$$

So $\Lambda \times L$ is an $(r,R)$-Delone set. $\square$

A convenient way to define the “stacking” map is by describing the C*-algebra of the product Delone set as a spatial tensor product. We note the following well-known lemma:

#### Lemma 4.5

Let $\mathcal {G}_1\rightrightarrows \Omega _1$ and $\mathcal {G}_2\rightrightarrows \Omega _2$ be étale groupoids. Let $\mathcal {G}_1\times \mathcal {G}_2$ be the product groupoid of $\mathcal {G}_1$ and $\mathcal {G}_2$. Then there is an isomorphism

$$\begin{aligned} \Phi :\textrm{C}^*(\mathcal {G}_1)\otimes \textrm{C}^*(\mathcal {G}_2)\xrightarrow {\sim }\textrm{C}^*(\mathcal {G}_1\times \mathcal {G}_2),\quad \Phi (f_1\otimes f_2)(\gamma _1,\gamma _2)\mathrel {:=}f_1(\gamma _1)f_2(\gamma _2), \end{aligned}$$

where $\otimes$ refers to the spatial tensor product.

#### Proof

This lemma is well-known, and we sketch a proof here.

Write $L^2(\mathcal {G}_1)$, $L^2(\mathcal {G}_2)$ and $L^2(\mathcal {G}_1\times \mathcal {G}_2)$ for the Hilbert C*-modules over $\textrm{C}_0(\Omega _1)$, $\textrm{C}_0(\Omega _2)$ and $\textrm{C}_0(\Omega _1\times \Omega _2)$ as in Section 3.1.1. We claim that $L^2(\mathcal {G}_1\times \mathcal {G}_2)$ is canonically isomorphic to the external tensor product Hilbert C*-module $L^2(\mathcal {G}_1)\otimes L^2(\mathcal {G}_2)$ under the identification

$$\begin{aligned} \Psi :\textrm{C}_0(\Omega _1)\otimes \textrm{C}_0(\Omega _2)\simeq \textrm{C}_0(\Omega _1\times \Omega _2),\quad \Psi (f\otimes g)(x,y)\mathrel {:=}f(x)\cdot g(y). \end{aligned}$$

To see this, let $f_1,f_2\in \textrm{C}_\textrm{c}(\mathcal {G}_1)$ and $g_1,g_2\in \textrm{C}_\textrm{c}(\mathcal {G}_2)$, Then their tensor products $f_1\otimes g_1, f_2\otimes g_2$ belong to $\textrm{C}_\textrm{c}(\mathcal {G}_1\times \mathcal {G}_2)$. Given $x\in \Omega _1$ and $y\in \Omega _2$, we have

$$\begin{aligned} \left\langle f_1,f_2\right\rangle (x)=\sum _{\gamma \in r^{-1}(x)}f_1(\gamma )f_2(\gamma ),\quad \left\langle g_1,g_2\right\rangle (y)=\sum _{\eta \in r^{-1}(y)}g_1(\eta ) g_2(\eta ), \end{aligned}$$

and

$$\begin{aligned} \left\langle f_1\otimes g_1,f_2\otimes g_2\right\rangle (x,y)&=\sum _{(\gamma ,\eta )\in r^{-1}(x,y)}(f_1\otimes g_1)(\gamma ,\eta )(f_2\otimes g_2)(\gamma ,\eta )\\&=\sum _{\gamma \in r^{-1}(x)}\sum _{\eta \in r^{-1}(y)}f_1(\gamma )g_2(\gamma )g_1(\eta )g_2(\eta ) \\&=\langle f_1,f_2\rangle (x)\cdot \langle g_1,g_2\rangle (y). \end{aligned}$$

Thus $L^2(\mathcal {G}_1\times \mathcal {G}_2)\simeq L^2(\mathcal {G}_1)\otimes L^2(\mathcal {G}_2)$ as Hilbert C*-modules over $\textrm{C}_0(\Omega _1\times \Omega _2)\simeq \textrm{C}_0(\Omega _1)\otimes \textrm{C}_0(\Omega _2)$. This induces an injective *-homomorphism (cf. ( [8], Section 13.5; [20], Chapter 4)):

$$\begin{aligned} \Phi :\mathbb {B}_{\textrm{C}_0(\Omega _1)}(L^2(\mathcal {G}_1))\otimes \mathbb {B}_{\textrm{C}_0(\Omega _2)}(L^2(\mathcal {G}_2))\rightarrow \mathbb {B}_{\textrm{C}_0(\Omega _1\times \Omega _2)}(L^2(\mathcal {G}_1\times \mathcal {G}_2)). \end{aligned}$$

In particular, for $f_1\in \textrm{C}_\textrm{c}(\mathcal {G}_1)$ and $f_2\in \textrm{C}_\textrm{c}(\mathcal {G}_2)$, viewed as convolution operators on $L^2(\mathcal {G}_1)$ and $L^2(\mathcal {G}_2)$ via their regular representations $\pi _i:\textrm{C}^*(\mathcal {G}_i)\rightarrow \mathbb {B}_{\textrm{C}_0(\Omega _i)}(\mathcal {G}_i)$ for $i=1,2$, then $\Phi$ sends $f_1\otimes f_2$ to the convolution operator $f_1\otimes f_2\in \textrm{C}_\textrm{c}(\mathcal {G}_1\times \mathcal {G}_2)$ on $L^2(\mathcal {G}_1\times \mathcal {G}_2)$. Passing to the norm closure, we find that $\Phi$ maps $\textrm{C}^*(\mathcal {G}_1)\otimes \textrm{C}^*(\mathcal {G}_2)$ into $\textrm{C}^*(\mathcal {G}_1\times \mathcal {G}_2)$, and has dense range as $\textrm{C}_\textrm{c}(\mathcal {G}_1\times \mathcal {G}_2)$ is dense in $\textrm{C}^*(\mathcal {G}_1\times \mathcal {G}_2)$. Thus $\Phi$ is both injective and has dense range, hence a *-isomorphism

$$\begin{aligned} \textrm{C}^*(\mathcal {G}_1)\otimes \textrm{C}^*(\mathcal {G}_2)\xrightarrow {\sim }\textrm{C}^*(\mathcal {G}_1\times \mathcal {G}_2). \end{aligned}$$

$$\square $$

#### Corollary 4.6

There is a canonical isomorphism

$$\begin{aligned} \textrm{C}^*(\mathcal {G}_{\Lambda \times L})\simeq \textrm{C}^*(\mathcal {G}_\Lambda )\otimes \textrm{C}^*(\mathcal {G}_L). \end{aligned}$$

#### Proof

By Lemma 4.5, it suffices to prove that the étale groupoids $\mathcal {G}_{\Lambda \times L}$ and $\mathcal {G}_{\Lambda }\times \mathcal {G}_L$ are isomorphic.

We claim that $\Omega _{\Lambda \times L}\simeq \Omega _\Lambda \times \Omega _L$ are isomorphic as $\mathbb {R}^{m+n}$-spaces. We have

$$\begin{aligned} \Lambda \times L\in \operatorname {Del}_{(r_\Lambda ,R_\Lambda )}(\mathbb {R}^m)\times \operatorname {Del}_{(r_L,R_L)}(\mathbb {R}^n). \end{aligned}$$

The translation action of $\mathbb {R}^{p+q}=\mathbb {R}^p\times \mathbb {R}^q$ preserves $\operatorname {Del}_{(r_\Lambda ,R_\Lambda )}(\mathbb {R}^p)\times \operatorname {Del}_{(r_L,R_L)}(\mathbb {R}^q)$. By Proposition 3.4, both $\operatorname {Del}_{(r_\Lambda ,R_\Lambda )}(\mathbb {R}^m)\times \operatorname {Del}_{(r_L,R_L)}(\mathbb {R}^n)$ are closed, hence their product is a closed subset of $\mathcal {M}(\mathbb {R}^{p+q})$. Since the product of the orbits of $\Lambda$ and $L$ is contained in $\operatorname {Del}_{(r_\Lambda ,R_\Lambda )}(\mathbb {R}^p)\times \operatorname {Del}_{(r_L,R_L)}(\mathbb {R}^q)$, we conclude that $\Omega _{\Lambda \times L}$, $\Omega _\Lambda \times \Omega _L$ are contained in it. Hence, $\Omega _{\Lambda \times L}\subseteq \Omega _\Lambda \times \Omega _L$ as it is the smallest closed subset in $\operatorname {Del}_{(r_\Lambda ,R_\Lambda )}(\mathbb {R}^p)\times \operatorname {Del}_{(r_L,R_L)}(\mathbb {R}^q)$, which contains the product of the joint orbits of $\Lambda$ and $L$.

We claim that $\Omega _{\Lambda \times L}\supseteq \Omega _\Lambda \times \Omega _L$ as well. Let $(\omega ,\ell )\in \Omega _\Lambda \times \Omega _L$, then there exists nets $(x_\alpha )_{\alpha \in A}\subseteq \mathbb {R}^p$ and $(a_\beta )_{\beta \in B}\subseteq \mathbb {R}^q$ such that

$$\begin{aligned} \Lambda +x_\alpha \rightarrow \omega \quad \text {and}\quad L+a_\beta \rightarrow \ell \quad \text {in the weak}  ^*\text {-topology.} \end{aligned}$$

Then the net $(x_\alpha ,a_\beta )_{\alpha \times \beta \in A\times B}$, where $A\times B$ carries the lexicographic order, satisfies

$$\begin{aligned} (\Lambda +x_\alpha ,L+a_\beta )_{(\alpha ,\beta )\in A\times B}\rightarrow (\omega ,\ell )\quad \text {in the weak} ^*\text {-topology}. \end{aligned}$$

Then we conclude that $\Omega _{\Lambda \times L}\simeq \Omega _\Lambda \times \Omega _L$ as $\mathbb {R}^{p+q}$-spaces.

As a consequence, the action groupoids $\Omega _{\Lambda \times L}\rtimes \mathbb {R}^{p+q}$ and $(\Omega _{\Lambda }\times \Omega _L)\rtimes \mathbb {R}^{p+q}$ are isomorphic topological groupoids, and have homeomorphic abstract transversals $\Omega _0^{\Lambda \times L}$ and $\Omega _0^{\Lambda }\times \Omega _0^L$. This implies an isomorphism of étale groupoids

$$\begin{aligned} \mathcal {G}_{\Lambda \times L}\simeq \mathcal {G}_\Lambda \times \mathcal {G}_L,\quad (\mu ,z)\mapsto ((\mu _\Lambda ,x),(\mu _L,a)) \end{aligned}$$

where $\mu _\Lambda$ and $\mu _L$ are the images of $\mu$ under the coordinate projections $\mathbb {R}^p\times \mathbb {R}^q\rightarrow \mathbb {R}^p$ and $\mathbb {R}^p\times \mathbb {R}^q\rightarrow \mathbb {R}^q$. This finishes the proof. $\square$

Corollary 4.6 allows us to define a *-homomorphism between groupoid C*-algebras. Since $\mathcal {G}_L$ is an étale groupoid with compact unit space, its C*-algebra $\textrm{C}^*(\mathcal {G}_L)$ has a unit, given by the constant function $1_{\Omega _0^L}$ on the unit space. Define

4.7 $$\begin{aligned} \varphi ^\textrm{Gpd}:\textrm{C}^*(\mathcal {G}_\Lambda )\rightarrow \textrm{C}^*(\mathcal {G}_\Lambda )\otimes \textrm{C}^*(\mathcal {G}_L),\quad \varphi ^\textrm{Gpd}(f)\mathrel {:=}f\otimes 1_{\Omega _0^L}. \end{aligned}$$

Then $\varphi ^\textrm{Gpd}$ gives a *-homomorphism $\textrm{C}^*(\mathcal {G}_\Lambda )\rightarrow \textrm{C}^*(\mathcal {G}_{\Lambda \times L})$ under the isomorphism $\textrm{C}^*(\mathcal {G}_{\Lambda \times L})\simeq \textrm{C}^*(\mathcal {G}_\Lambda )\otimes \textrm{C}^*(\mathcal {G}_L)$ in Corollary 4.6. We write $\varphi ^\textrm{Gpd}_N:\mathbb {M}_N(\textrm{C}^*(\mathcal {G}_\Lambda ))\rightarrow \mathbb {M}_N(\textrm{C}^*(\mathcal {G}_{\Lambda \times L}))$ for its entrywise extension to matrix algebras.

Now we pass to Roe C*-algebras. Let $\omega \in \Omega _0^\Lambda$ and $\ell \in \Omega _0^L$. Define their Roe C*-algebras $\textrm{C}^*_\textrm{Roe}(\omega )$ and $\textrm{C}^*_\textrm{Roe}(\omega \times \ell )$ using their standard ample modules $\ell ^2(\omega ,\mathcal {K})$ and $\ell ^2(\omega \times \ell ,\mathcal {K})$. Let

4.8 $$\begin{aligned} \varphi ^\textrm{Roe}:\mathbb {B}(\ell ^2(\omega ,\mathcal {K}))\rightarrow \mathbb {B}(\ell ^2(\omega \times \ell ,\mathcal {K})),\quad T\mapsto T\otimes \operatorname {id}_{\ell ^2(\ell )}. \end{aligned}$$

ThenSo $\varphi ^\textrm{Roe}(T)$ is locally compact or controlled if and only if $T$ is locally compact or controlled (cf. Example 3.18). Thus $\varphi ^\textrm{Roe}$ maps $\textrm{C}^*_\textrm{Roe}(\omega )$ into $\textrm{C}^*_\textrm{Roe}(\omega \times \ell )$.

#### Lemma 4.9

The map $\varphi ^\textrm{Roe}:\textrm{C}^*_\textrm{Roe}(\omega )\rightarrow \textrm{C}^*_\textrm{Roe}(\omega \times \ell )$ induces zero maps

$$\begin{aligned} \varphi ^\textrm{Roe}_*:\operatorname {KK}(\textrm{C}\ell _{n,0},\textrm{C}^*_\textrm{Roe}(\omega ))\rightarrow \operatorname {KK}(\textrm{C}\ell _{n,0},\textrm{C}^*_\textrm{Roe}(\omega \times \ell )) \end{aligned}$$

for all $$ n $$.

#### Proof

Let

$$\begin{aligned} \ell _+\mathrel {:=}\ell \cap (\mathbb {R}^{q-1}\times \mathbb {R}_{\ge 0}),\quad \ell _-\mathrel {:=}\ell \cap (\mathbb {R}^{q-1}\times \mathbb {R}_{< 0}), \end{aligned}$$

then there are isometries

$$\begin{aligned} V_\pm :\ell ^2(\omega \times \ell _\pm ,\mathcal {K})\rightarrow \ell ^2(\omega \times \ell ,\mathcal {K}), \end{aligned}$$

which induce a diagonal embedding

$$\begin{aligned} \mathbb {B}(\ell ^2(\omega \times \ell _+,\mathcal {K}))\oplus \mathbb {B}(\ell ^2(\omega \times \ell _-,\mathcal {K}))&\rightarrow \mathbb {B}(\ell ^2(\omega \times \ell ,\mathcal {K})),\\ (T_+,T_-)&\mapsto \operatorname {Ad}_{V_+}T_++\operatorname {Ad}_{V_-}T_-. \end{aligned}$$

Let $T\in \mathbb {B}(\ell ^2(\omega ,\mathcal {K}))$. Then $T\otimes \operatorname {id}_{\ell ^2(\ell )}=\operatorname {Ad}_{V_+}(T\otimes \operatorname {id}_{\ell ^2(\ell _+)}) +\operatorname {Ad}_{V_-}(T\otimes \operatorname {id}_{\ell ^2(\ell _-)})$. Therefore, the map $\varphi ^\textrm{Roe}$ agrees with the following composition:

$$\begin{aligned} \mathbb {B}(\ell ^2(\omega ,\mathcal {K}))\rightarrow \mathbb {B}(\ell ^2(\omega \times \ell _+,\mathcal {K}))\oplus \mathbb {B}(\ell ^2(\omega \times \ell _-,\mathcal {K}))\rightarrow \mathbb {B}(\ell ^2(\omega \times \ell ,\mathcal {K})),\\ T\mapsto \left( T\otimes \operatorname {id}_{\ell ^2(\ell _+)},T\otimes \operatorname {id}_{\ell ^2(\ell _-)}\right) \mapsto \operatorname {Ad}_{V_+}\left( T\otimes \operatorname {id}_{\ell ^2(\ell _+)}\right) +\operatorname {Ad}_{V_-}\left( T\otimes \operatorname {id}_{\ell ^2(\ell _-)}\right) . \end{aligned}$$

A similar argument as the lines below (4.8) shows that $T\mapsto (T\otimes \operatorname {id}_{\ell ^2(\ell _+)},T\otimes \operatorname {id}_{\ell ^2(\ell _-)})$ maps $\textrm{C}^*_\textrm{Roe}(\omega )$ into $\textrm{C}^*_\textrm{Roe}(\omega \times \ell _+)\oplus \textrm{C}^*_\textrm{Roe}(\omega \times \ell _-)$. Both spaces $\omega \times \ell _\pm$ are flasque (cf. [25], Definition 9.3). Hence, $\textrm{C}^*_\textrm{Roe}(\omega \times \ell _\pm )$ have vanishing K-theory by an Eilenberg swindle argument, cf. ( [25], Theorem 9.4). Thus $\varphi ^\textrm{Roe}_*$ vanishes as it factors through zero. $\square$

#### Theorem 4.10

(Stacked topological phases are weak) Let $\xi ^\textrm{Gpd}_{\omega \times \ell ,N}$ be the spectral triple over $\textrm{C}^*(\mathcal {G}_{\Lambda \times L})\otimes \mathbb {M}_N(\mathbb {R})$ defined in (3.13). Then for each $n$, its induced map

$$\begin{aligned} \operatorname {KK}(\textrm{C}\ell _{n,0},\textrm{C}^*(\mathcal {G}_{\Lambda \times L}))\rightarrow \operatorname {KK}(\textrm{C}\ell _{n,p+q},\mathbb {R}) \end{aligned}$$

vanishes on the image of

$$\begin{aligned} \varphi ^\textrm{Gpd}_*:\operatorname {KK}(\textrm{C}\ell _{n},\textrm{C}^*(\mathcal {G}_\Lambda ))\rightarrow \operatorname {KK}(\textrm{C}\ell _{n},\textrm{C}^*(\mathcal {G}_{\Lambda \times L})). \end{aligned}$$

#### Proof

We claim that the following diagram commutes: 
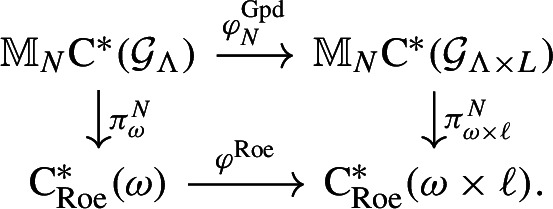


To see this, let $f\in \textrm{C}_\textrm{c}(\mathcal {G}_\Lambda )$ and $S\in \mathbb {M}_N(\mathbb {R})$. It follows from (4.7) and (4.8) that:holds for all $x,y\in \omega$ and $a,b\in \ell$. Therefore the maps $\varphi ^\textrm{Roe}\circ \pi _\omega ^N$ coincides with $\pi _{\omega \times \ell }^N\circ \varphi ^\textrm{Gpd}$ on all elements of the form $f\otimes S$, and hence for $\textrm{C}^*(\mathcal {G}_\Lambda )\otimes \mathbb {M}_N(\mathbb {R})$. That is, the diagram above commutes.

The commutative diagram above gives a commutative diagram in K-theory. This, together with the bottom left triangle of 4.2, gives the following commutative diagram: 
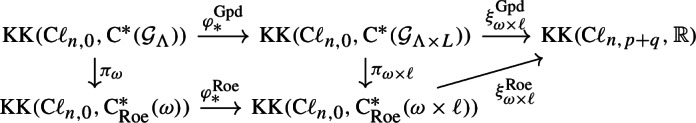


where $\xi ^\textrm{Gpd}_{\omega \times \ell }$ and $\xi ^\textrm{Roe}_{\omega \times \ell }$ are the corresponding position spectral triples over $\textrm{C}^*(\mathcal {G}_{\Lambda \times L})$ and $\textrm{C}^*_\textrm{Roe}(\omega \times \ell )$. It follows that the composition map $\xi ^\textrm{Gpd}_{\omega \times \ell }\circ \varphi _* =\xi ^\textrm{Roe}_{\omega \times \ell }\circ \varphi ^\textrm{Roe}_*\circ \pi _\omega$ factors through

$$\begin{aligned} \varphi ^\textrm{Roe}_*:\operatorname {KK}(\textrm{C}\ell _{n,0},\textrm{C}^*_\textrm{Roe}(\omega ))\rightarrow \operatorname {KK}(\textrm{C}\ell _{n,0},\textrm{C}^*_\textrm{Roe}(\omega \times \ell )), \end{aligned}$$

which vanishes by Lemma 4.9. Therefore $\xi ^\textrm{Gpd}_{\omega \times \ell }\circ \varphi ^\textrm{Gpd}_*$ must be the zero map. $\square$

#### Example 4.11

Let $\Lambda =\mathbb {Z}^d$ and $L=\mathbb {Z}$. Then $\mathcal {G}_\Lambda \simeq \mathbb {Z}^d$ and $\mathcal {G}_L\simeq \mathbb {Z}$ as topological groupoids. The unit spaces $\Omega _0^{\Lambda }$ and $\Omega _0^{\Lambda \times L}$ are both singletons. So there are unique localised regular representations $\pi :\textrm{C}^*(\mathbb {Z}^d)\rightarrow \textrm{C}^*_\textrm{Roe}(\mathbb {Z}^d)$ and $\textrm{C}^*(\mathbb {Z}^{d+1})\rightarrow \textrm{C}^*_\textrm{Roe}(\mathbb {Z}^{d+1})$. Let $N=1$. The *-homomorphism $\varphi ^\textrm{Gpd}_1:\textrm{C}^*(\mathbb {Z}^d)\rightarrow \textrm{C}^*(\mathbb {Z}^{d+1})$ maps an element in $\textrm{C}^*(\mathbb {Z}^{d+1})$ to its restriction to the first $d$-coordinates. Identify $\textrm{C}^*(\mathbb {Z}^d)$ with $\textrm{C}(\mathbb {T}^d)$ via Fourier transform, then the map $\varphi ^\textrm{Gpd}$ sends $f\in \textrm{C}(\mathbb {T}^d)$ to $f\otimes 1\in \textrm{C}(\mathbb {T}^d)\otimes \textrm{C}(\mathbb {T})=\textrm{C}(\mathbb {T}^{d+1})$. The map $\varphi ^\textrm{Gpd}$ is induced by the injective group homomorphism

$$\begin{aligned} \mathbb {Z}^d\rightarrow \mathbb {Z}^{d+1},\quad x\mapsto (x,0), \end{aligned}$$

and its image belongs to the kernel of the map

$$\begin{aligned} \operatorname {KK}(\textrm{C}\ell _{n,0},\textrm{C}^*(\mathbb {Z}^d))\rightarrow \operatorname {KK}(\textrm{C}\ell _{n,0},\textrm{C}^*_\textrm{Roe}(\mathbb {Z}^d)), \end{aligned}$$

which yields ( [12], Proposition 10).

## Data Availability

Data sharing is not applicable in this study as no new data were created or analysed.

## References

[1] Arens, R.F., Kaplansky, I.: Topological representation of algebras. Trans. Amer. Math. Soc. **63**, 457–481 (1948)

[2] Anderson, J.E., Putnam, I.F.: Topological invariants for substitution tilings and their associated -algebras. Ergodic Theory Dynam. Systems **18**(3), 509–537 (1998)

[3] Bourne, C., Carey, A.L., Lesch, M., Rennie, A.: The KO-valued spectral flow for skew-adjoint Fredholm operators. J. Topol. Anal. **14**(2), 505–556 (2022)

[4] Bourne, C., Carey, A.L., Rennie, A.: A non-commutative framework for topological insulators. Rev. Math. Phys. **28**(2), 1650004, 51 (2016) MR 3484317

[5] Bellissard, J.: K-theory of -algebras in solid state physics. Statistical mechanics and field theory: mathematical aspects (Groningen, 1985) **257**, 99–156 (1986)

[6] Bellissard, J., Herrmann, D. J. L., Zarrouati, M., Hulls of aperiodic solids and gap labeling theorems, Directions in mathematical quasicrystals, 207–258 (2000) MR 1798994

[7] Bourne, C., Kellendonk, J., Rennie, A.: The Cayley transform in complex, real and graded K-theory. Internat. J. Math. **31**(9), 2050074 (2020)

[8] Blackadar, B.: K-theory for operator algebras, 2, p. 5. Mathematical Sciences Research Institute Publications, Cambridge University Press, Cambridge, (1998) MR 1656031

[9] Bourne, C., Mesland, B.: Index theory and topological phases of aperiodic lattices. Ann. Henri Poincaré **20**(6), 1969–2038 (2019)

[10] Bourne, C., Prodan, E.: Non-commutative Chern numbers for generic aperiodic discrete systems. J. Phys. A **51**(23), 235202 (2018)

[11] Bellissard, J., van Elst, A., Schulz-Baldes, H.: The noncommutative geometry of the quantum Hall effect. J. Math. Phys. **35**(10), 5373–5451 (1994)

[12] Ewert, E.E., Meyer, R.: Coarse geometry and topological phases. Comm. Math. Phys. **366**(3), 1069–1098 (2019)

[13] Higson, N., Roe, J., Yu, G.: A coarse Mayer-Vietoris principle. Math. Proc. Cambridge Philos. Soc. **114**(1), 85–97 (1993)

[14] Kasparov, G.: Topological invariants of elliptic operators. I. K-homology,. Math. USSR-Izv. **9**(4), 751–792 (1975)

[15] Kasparov, G.: The operator K-functor and extensions of -algebras. Izv. Akad. Nauk SSSR Ser. Mat. **44**(3), 571–636 (1980)

[16] Kasparov, G.: Equivariant KK-theory and the Novikov conjecture. Invent. Math. **91**(1), 147–201 (1988)

[17] Kellendonk, J.: On the -algebraic approach to topological phases for insulators. Ann. Henri Poincaré **18**(7), 2251–2300 (2017)

[18] Khoshkam, M., Skandalis, G.: Regular representation of groupoid -algebras and applications to inverse semigroups. J. Reine Angew. Math. **546**, 47–72 (2002)

[19] Kubota, Y.: Controlled topological phases and bulk-edge correspondence. Comm. Math. Phys. **349**(2), 493–525 (2017)

[20] Lance, E. C.: Hilbert -modules, London Mathematical Society Lecture Note Series, Cambridge University Press, Cambridge, 210 (1995). A toolkit for operator algebraists, MR 1325694

[21] Lord, S., Rennie, A., Várilly, J.C.: Riemannian manifolds in noncommutative geometry. J. Geom. Phys. **62**(7), 1611–1638 (2012)

[22] Mesland, B.: Groupoid cocycles and K-theory. Münster J. Math. **4**, 227–249 (2011)

[23] Mesland, B., Prodan, E.: A groupoid approach to interacting fermions. Comm. Math. Phys. **394**(1), 143–213 (2022)

[24] Muhly, P.S., Renault, J.N., Williams, D.P.: Equivalence and isomorphism for groupoid -algebras. J. Operator Theory **17**(1), 3–22 (1987)

[25] Roe, J.: Index theory, coarse geometry, and topology of manifolds, CBMS Regional Conference Series in Mathematics, Published for the Conference Board of the Mathematical Sciences, Washington, p. 90. DC; by the American Mathematical Society, Providence, RI (1996) MR 1399087

[26] Špakula, J.: K-theory of uniform Roe algebras, ProQuest LLC, Ann Arbor, MI, Thesis (Ph.D.)–Vanderbilt University, (2008) MR 2712872

[27] Sadun, L., Williams, R.F.: Tiling spaces are Cantor set fiber bundles. Ergodic Theory Dynam. Systems **23**(1), 307–316 (2003)

[28] Thiang, G.C.: On the K-theoretic classification of topological phases of matter. Ann. Henri Poincaré **17**(4), 757–794 (2016)

[29] Willett, R., Yu, G.: Higher index theory, Cambridge Studies in Advanced Mathematics, Cambridge University Press, Cambridge, 189 (2020) MR 4411373

